# Insight into glucocorticoid receptor signalling through interactome model analysis

**DOI:** 10.1371/journal.pcbi.1005825

**Published:** 2017-11-06

**Authors:** Emyr Bakker, Kun Tian, Luciano Mutti, Constantinos Demonacos, Jean-Marc Schwartz, Marija Krstic-Demonacos

**Affiliations:** 1 Biomedical Research Centre, School of Environment and Life Sciences, University of Salford, Salford, United Kingdom; 2 Faculty of Biology, Medicine and Health, University of Manchester, Manchester, United Kingdom; University of Chicago, UNITED STATES

## Abstract

Glucocorticoid hormones (GCs) are used to treat a variety of diseases because of their potent anti-inflammatory effect and their ability to induce apoptosis in lymphoid malignancies through the glucocorticoid receptor (GR). Despite ongoing research, high glucocorticoid efficacy and widespread usage in medicine, resistance, disease relapse and toxicity remain factors that need addressing. Understanding the mechanisms of glucocorticoid signalling and how resistance may arise is highly important towards improving therapy. To gain insight into this we undertook a systems biology approach, aiming to generate a Boolean model of the glucocorticoid receptor protein interaction network that encapsulates functional relationships between the GR, its target genes or genes that target GR, and the interactions between the genes that interact with the GR. This model named GEB052 consists of 52 nodes representing genes or proteins, the model input (GC) and model outputs (cell death and inflammation), connected by 241 logical interactions of activation or inhibition. 323 changes in the relationships between model constituents following *in silico* knockouts were uncovered, and steady-state analysis followed by cell-based microarray genome-wide model validation led to an average of 57% correct predictions, which was taken further by assessment of model predictions against patient microarray data. Lastly, semi-quantitative model analysis via microarray data superimposed onto the model with a score flow algorithm has also been performed, which demonstrated significantly higher correct prediction ratios (average of 80%), and the model has been assessed as a predictive clinical tool using published patient microarray data. In summary we present an *in silico* simulation of the glucocorticoid receptor interaction network, linked to downstream biological processes that can be analysed to uncover relationships between GR and its interactants. Ultimately the model provides a platform for future development both by directing laboratory research and allowing for incorporation of further components, encapsulating more interactions/genes involved in glucocorticoid receptor signalling.

## Author summary

Here we present modelling of the glucocorticoid receptor (GR) signalling network. The GR is the effector for a class of drugs known as corticosteroids, which are widely used in medicine for their anti-inflammatory effects and ability to induce apoptosis in leukaemic cells. However, side effects, treatment-related toxicity and glucocorticoid resistance remain and therefore increased understanding of the glucocorticoid receptor mechanism of action may improve therapeutic outcomes. The GEB052 model presented herein has been used to generate predictions for how the network is altered between glucocorticoid-sensitive and glucocorticoid-resistant scenarios, and these predictions have been verified using published gene expression data from established cell lines (for both qualitative and semi-quantitative analysis). The model has also been preliminarily assessed as a predictive clinical tool by correlating model predictions with clinical outcomes of thirteen leukaemia patients. Thus, the GEB052 model demonstrates successful modelling to understand GR function. GEB052 provides accurate predictions and has indicated potential routes through which glucocorticoid resistance may arise. The work presented herein thus demonstrates a proof-of-principle of this modelling approach to furthering GR research, and provides insight into potential mechanisms of corticosteroids resistance.

## Introduction

Glucocorticoids (GCs) steroid hormones released from the adrenal cortex as part of the stress response play an important role in a variety of bodily processes such as inflammation, immunity, and numerous metabolic processes [1–3]. Their varied effects allow for their clinical application in numerous diseases, particularly for their potent anti-inflammatory and immunosuppressive effects to treat diseases such as arthritis [4, 5]. GCs are also prescribed for the treatment of lymphoid cancers, as they selectively induce cell death in leukocytes [6–8], highlighting the tissue specificity of their action and the need for further research into GC signalling.

GCs exert their effects through the glucocorticoid receptor (GR) which is an intracellular cytoplasmic receptor which, in the absence of a ligand, is part of a complex with chaperones such as heat-shock protein 90 [9]. Following ligand binding, GR dissociates from this complex and translocates to the nucleus where it regulates the expression of its target genes as an active transcription factor [10, 11]. Numerous factors control GR activity, including phosphorylation status [12], targeting to protein degradation pathways [13] and interaction with cofactors [14].

Clinically, synthetic GCs such as dexamethasone are used due to their higher potency and stability. Whilst GCs have achieved significant therapeutic outcomes, resistance to treatment and side-effects both remain an issue. Defective GR expression, Bcl-2 overexpression, and other aberrant signalling may contribute to glucocorticoid resistance [6, 15, 16]. Increased knowledge into the details of GR signalling may allow for the development of novel therapeutics and identification of resistance factors. Although high-throughput methodologies have provided insight into GR signalling [6], there remains a need to properly integrate large datasets in a cohesive manner.

Systems biology aims to accurately represent biological phenomena by constructing integrative models of molecular components and their interactions. Some models are quantitatively precise and require measurement of biological kinetic data, though they are often of a smaller scale, aiming to precisely model a particular subset of interactions. This approach has been applied to glucocorticoid research in numerous ways, such as the development of models of GR/c-Jun/Erg (Ets-related gene) crosstalk [17]. The models of GR/c-Jun/Erg confirmed known interaction phenomena but also identified Erg as a putative marker for glucocorticoid resistance [17]. Although such models provide useful insight, they are both time-consuming and resource-expensive to create due to the required biological data. Boolean modelling on the contrary allows for the generation of large-scale models that provide a qualitative overview of the behaviour of an entire network [18, 19]. In these cases, interactions and molecular levels are simplified to ON or OFF binary values, removing the need to know exact rate and kinetic equations thus reducing computational demand [20].

We have previously demonstrated that Boolean modelling may be successfully applied to cancer research through generating the PKT206 model of the p53 interactome [18] which has revealed novel mechanisms of p53 signalling and how this may be disrupted following loss of p53 function. Correct prediction rates reached 71% for the model, signifying the strength of this approach [18]. An expanded p53 interactome was later developed to more accurately model the signalling phenomena [19].

To overcome the qualitative nature of the Boolean modelling approach algorithms utilising microarray and/or ChIP-seq data have been developed such as the signal transduction score flow algorithm (STSFA) which analyses Boolean models in a semi-quantitative manner [21]. This algorithm has been applied to the original PKT206 model [22], which demonstrated improved predictive power over the original model analysis. Thus, application of this or similar algorithms represents a way to improve model accuracy through its semi-quantitative nature.

The aim of this research was to develop a Boolean model for the GR interaction network similar to the p53 interactomes [18, 19]. The model (GEB052: **G**lucocorticoid receptor model by **E**myr **B**akker, consisting of **52** nodes) contains 241 interactions. Nodes represent genes/proteins or inputs (glucocorticoid)/outputs (cell death and inflammation). CellNetAnalyzer [23] has been used for *in silico* analysis. Boolean model performance was assessed via comparison to microarray data [18] which demonstrated up to 60.4% of predictions depending on microarray data used for validation (average 57%) as correct, whilst STSFA analysis indicated a correct prediction rate of 80.1%. Using microarray data from thirteen leukaemia patients the model has been assessed as a predictive clinical tool. This report demonstrates the applicability of this modelling approach to nuclear receptor research, with the overarching aim being to eventually create models in a tissue-type, disease-specific and patient-centred manner.

## Results

### GEB052 network generation

**Fig 1:**
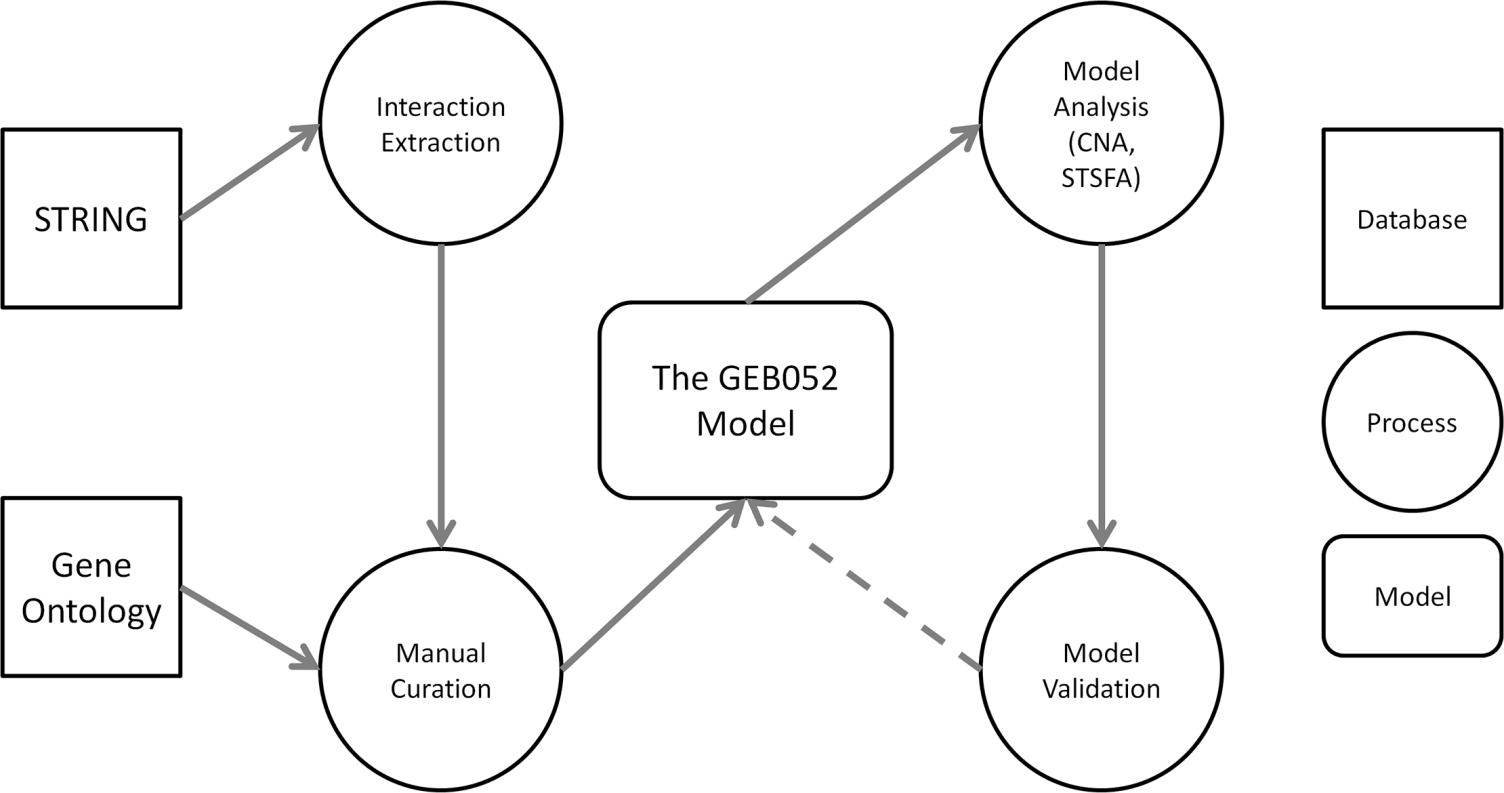
Flow chart demonstrating the workflow of GEB052 model construction and analysis. Database files were downloaded from the STRING website and interactions for proteins of interest were extracted. Extensive manual curation of predicted interactions was performed via literature searching, and the model was linked to biological outputs (cell death and inflammation) through manual curation of Gene Ontology records. CellNetAnalyzer (CNA) and the Signal Transduction Score Flow Algorithm (STSFA) were used for model analysis, with model predictions being verified via microarray data. The dashed line from Model Validation to The GEB052 Model represents validation and potential model refinement through assessment of model predictions.

The GEB052 model was built via a similar workflow to the PKT206 model [18] (Fig 1). STRING (Search Tool for the Retrieval of Interacting Genes/Proteins) is a database that provides information on functional associations between proteins, and thus this database represented a starting point for the interactions to be included within the model [24, 25]. Nodes within this model represent genes (or their associated proteins) and inputs/outputs such as the glucocorticoid and cell death and inflammation respectively. Model edges represent activation or inhibition relationships between model constituents.

To ensure consistency and cohesiveness of the model for the primary layer (proteins interacting with the GR), proteins interacting in a highly indirect manner (i.e. through multiple steps and proteins) were excluded during curation. The curation evidence used for cofactors would indicate either the stimulatory or inhibitory effect of that cofactor on the GR, or a report demonstrating that the cofactor in question was a GR coactivator or corepressor. For the curation of the second layer (interactions between the proteins within the primary layer) the “intermediary rule” was applied. This rule covered cases for which literature curation indicated that despite STRING listing a direct link between Protein 1 and Protein 2 (both of which interact with the GR individually), this regulation actually occurred through an intermediary protein (i.e. Protein 1 -> Intermediary Protein -> Protein 2). In these cases, if the intermediary protein was present within the primary layer then the reactions would be listed as proceeding through the intermediary protein (i.e. Protein 1 -> Intermediary Protein -> Protein 2), provided no additional evidence of a direct relationship of Protein 1 -> Protein 2 was observed. In cases where the intermediary protein did not exist within the primary layer, the reaction instead was put as a direct Protein 1 -> Protein 2 to reduce redundancy.

In numerous cases, multiple proteins were combined as one node within the model. This was due to either the proteins forming a heterodimer or proteins from the same family being grouped together. These nodes and their constituents can be seen in the S1 Text file.

Following completion of the second layer, the model was connected to cell death and inflammation as two outputs through Gene Ontology. The full curation tables for the model (detailing the mode of interaction and at least one PubMed ID linking to a paper verifying the interaction) for the primary layer, second layer, and link to outputs can be seen in the S1 Text file.

### GEB052 model structure

**Fig 2:**
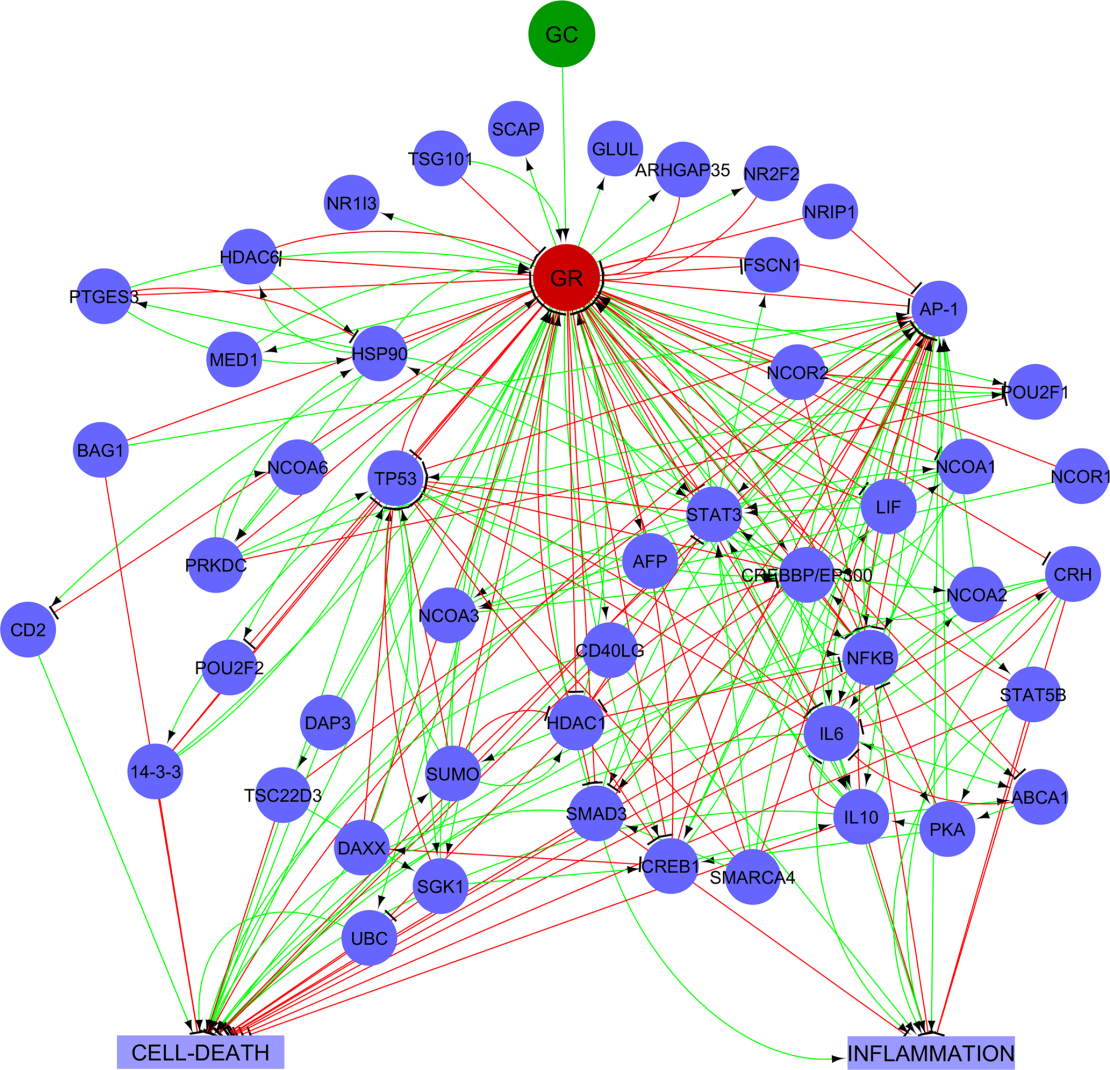
The GEB052 model. Nodes are represented by small blue circles, with the exception of the Input Node (GC) which is a green circle. The red circle represents the central node (the GR). Cell death and inflammation, the two model outputs, are shown in blue squares. Inhibitory edges are shown as red closed arrows whilst activation edges are shown as green open arrows.

**Fig 3:**
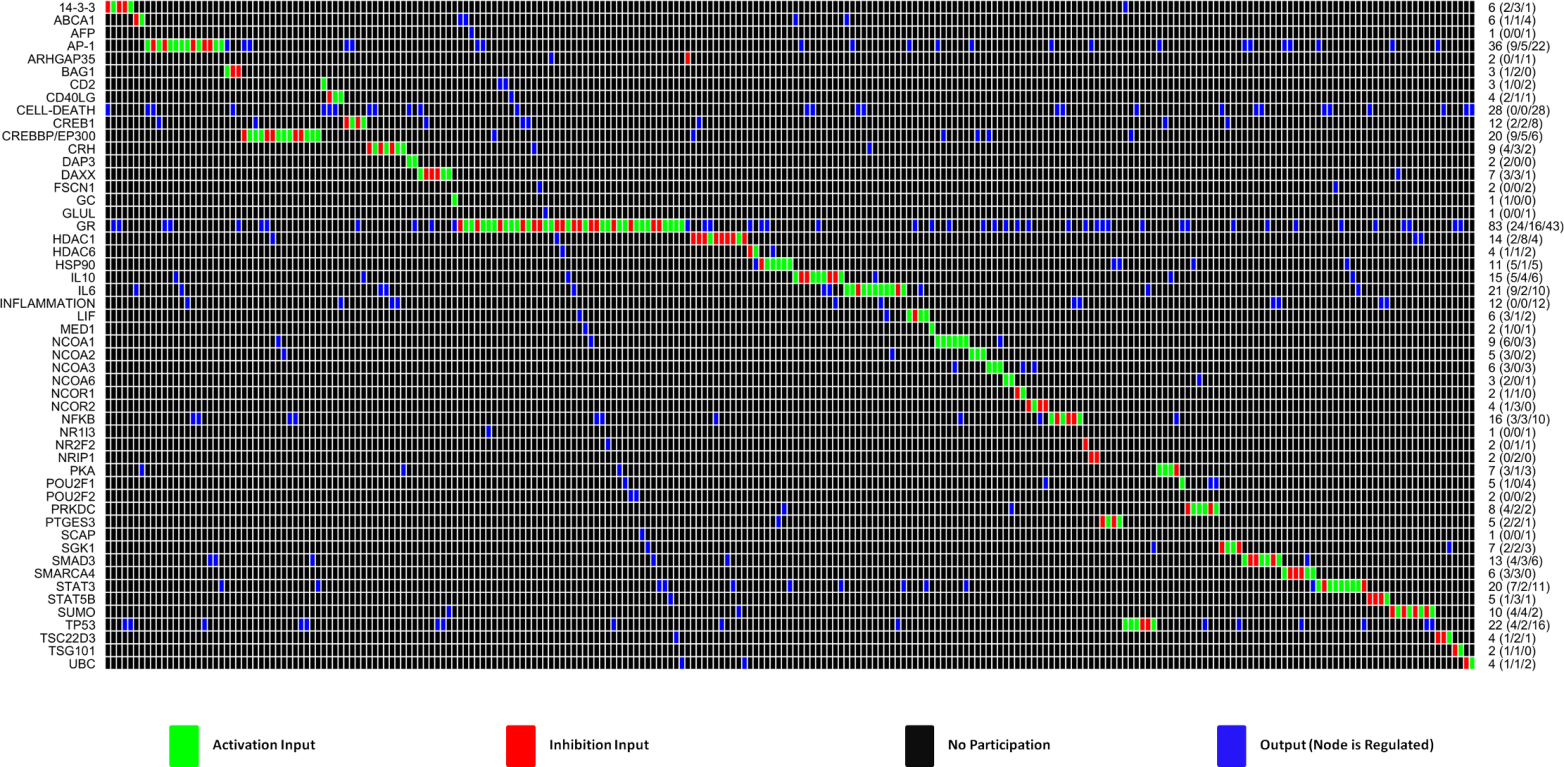
Interaction matrix for GEB052 model. Figure adapted from the CNA-generated interaction matrix. The right-hand y-axis shows the number of reactions that each node is involved in, whilst the left-hand y axis shows the nodes present within the model. For the right-hand axis, numbers in brackets are equal to the number of nodes it activates, the number of nodes it inhibits, and the number of nodes it is regulated by. All model nodes for all model edges are assigned a value in the interaction matrix. Black is equivalent to no participation, whilst blue means the node is affected (i.e. regulated) by the interaction. Red means the node has an inhibition input whilst green means the node has a stimulatory input.

The GEB052 model (Fig 2) consists of 52 nodes (proteins, inputs, outputs) connected by 241 logical interactions of activation or inhibition. Although the visualisation shown in Fig 2 is useful for providing an overview, it can be difficult to follow individual reactions in this detailed overview. As a complement to the full visualisation shown above, an interaction matrix (generated in CNA) is shown in Fig 3.

Feedback loops within biological networks are essential to maintain network integrity [18]. The GEB052 model contained 64 two step (i.e. Protein 1 -> Protein 2 -> Protein 1) loops, 26 of which (40.6%) involved the GR. This thus highlights the obvious centrality and importance of the GR within the network. Only two-step feedback loops are considered for examination as feedback loops may otherwise consist of numerous steps which would complicate analysis [18].

**Fig 4:**
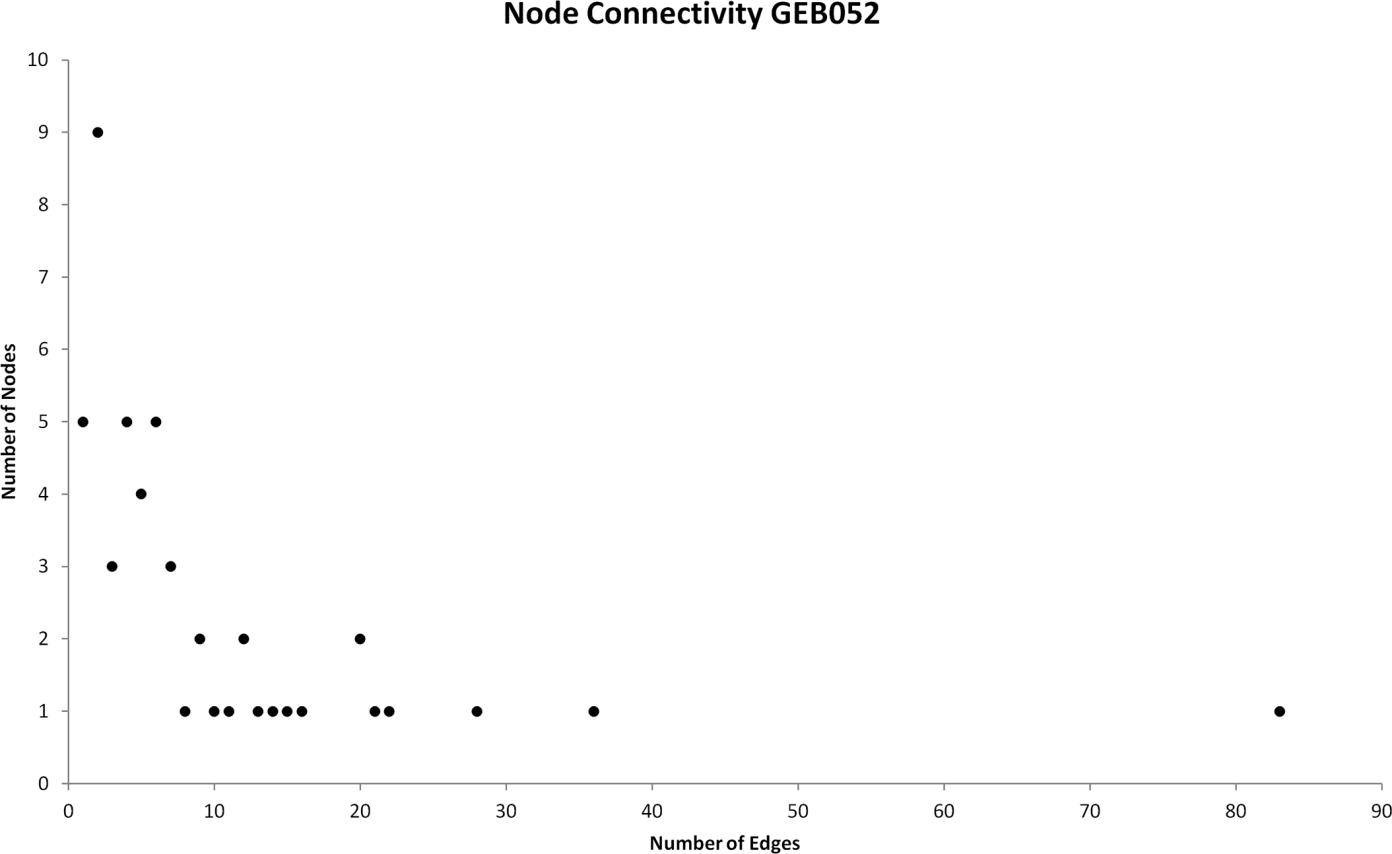
Node connectivity of GEB052 model. The number of edges interacting with the node is shown on the y-axis whilst the number of nodes with that degree of connectivity is shown on the x-axis.

In addition to feedback loop assessment, the degree (node connectivity) distribution was assessed (Fig 4). Excluding cell death and inflammation, six nodes demonstrated a very high level of connectivity (twenty or more edges). On the far right of Fig 4 is the GR, with a degree of 83. In addition to the GR, other nodes showing a very high degree include: AP-1 (36 edges); CREBBP/EP300 (20 edges); IL6 (21 edges); STAT3 (20 edges) and TP53 (22 edges). Other nodes exhibiting a high degree (ten or more edges) include: CREB1 (12 edges); HDAC1 (14 interactions); HSP90 (11 edges); IL10 (15 edges); NFKB (16 edges); SMAD3 (13 edges) and SUMO (10 edges). Other nodes (n = 37) exhibited a lower degree, possessing less than ten edges. Table 1 summarises the degree range observed within the model.

**Table 1:** Node connectivity of GEB052 model.

Node Degree Range	Number of Nodes	Percentage of Total Nodes
Connectivity > 80	1	1.9%
10 ≤ Connectivity ≤ 80	14	26.9%
0 < Connectivity < 10	37	71.2%

Understanding the node connectivity within the model was crucial to the choice of which nodes would be selected for *in silico* knockout analysis, as previous studies have focussed on *in silico* knockouts for only the most highly connected nodes [18, 19].

### Dependency and *in silico* knockout analysis of GEB052 model

**Fig 5:**
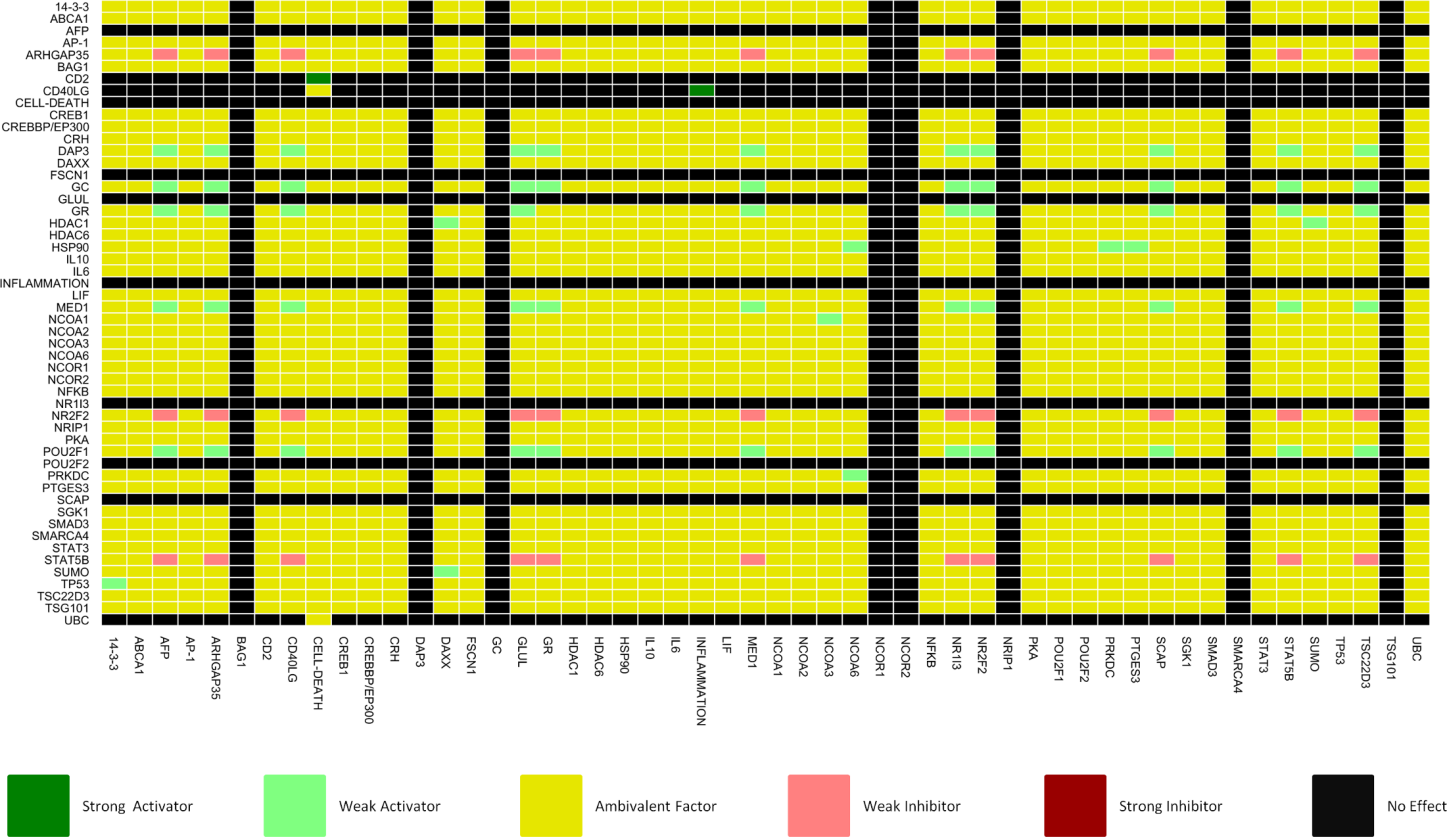
Dependency matrix for GEB052 model. Dependencies show the effect of the node on the y-axis on the node on the x-axis.

CNA is capable of generating a dependency matrix which, by taking into account all of the signalling pathways present within the model, is able to determine the overall relationships from one node to another. Six types of dependencies are available: no effect; ambivalent (stimulatory and inhibitory influence); weak inhibitor; weak activator; strong inhibitor and strong activator. Fig 5 shows the visualised dependency matrix for the full GEB052 model. It is apparent from examination of Fig 5 that the majority of dependencies are ambivalent factors. This observation correlates with the large number of feedback loops, as the highly integrated signalling within the model can lead to multiple signalling paths between model nodes, both positive and negative. As ambivalent dependencies are those most likely to change following *in silico* knockouts, the high number of ambivalent dependencies represents a good starting point for downstream analysis.

In total, 2704 (52*52) dependencies were observed within the GEB052 model: 896 of these were of no effect; 1710 were ambivalent; 33 were weak inhibitors; 63 were weak activators; 2 were strong activators and there were no strong inhibitors. To characterise how relationships are altered after perturbation to the model (mimicking potential mutations *in vivo*), each of the highly connected nodes (≥10 interactions, excepting model outputs) was deleted from the model and a dependency matrix generated, with the results shown in Table 2.

**Table 2:** Dependency matrix alterations following *in silico* knockout analysis.

Scenario	Number of Each Dependency						
	No Effect	Ambivalent	Weak Inhibitor	Weak Activator	Strong Inhibitor	Strong Activator	Total
**Full Model**	896	1710	33	63	0	2	2704
**AP-1 KO**	877	1581	66	75	0	2	2601
**CREB1 KO**	877	1626	33	63	0	2	2601
**CREBBP/** **EP300 KO**	877	1576	61	85	0	2	2601
**GR KO**	1602	955	5	35	1	3	2601
**HDAC1 KO**	953	1541	36	65	0	6	2601
**HSP90 KO**	993	1481	53	68	0	6	2601
**IL6 KO**	877	1607	43	72	0	2	2601
**IL10 KO**	877	1626	33	63	0	2	2601
**NFKB KO**	877	1626	33	63	0	2	2601
**SMAD3 KO**	877	1626	33	63	0	2	2601
**STAT3 KO**	877	1574	63	85	0	2	2601
**SUMO KO**	917	1589	33	60	0	2	2601
**TP53 KO**	917	1579	36	67	0	2	2601

**Fig 6:**
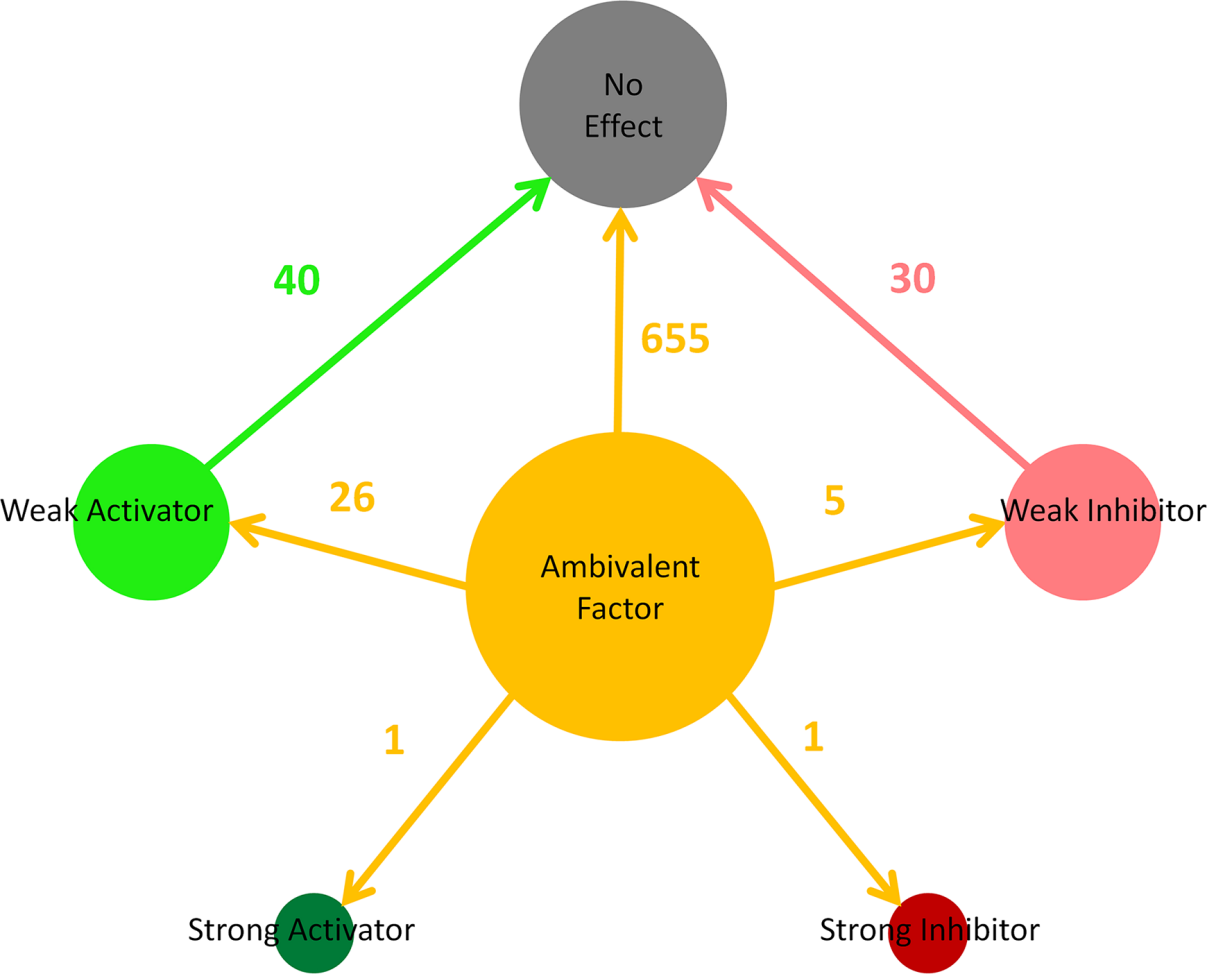
Dependency alteration distribution following an *in silico* GR knockout. This figure shows the alteration of dependencies following the removal of the GR node from the GEB052 model. Ambivalent dependencies are represented by a yellow circle, whilst weak activators and inhibitors are represented by a light green and pink circle respectively. The dark green circle represents strong activators, whilst the dark red circle represents strong inhibitors. No effect dependencies are represented by the dark grey circle.

For each knockout scenario, a total of 2601 (51*51) dependencies was observed and as expected due its centrality within the model, the removal of the GR had the most significant effects on the dependencies (Fig 6). The majority of dependency alterations were from ambivalent factors to no effect, which is consistent with the high connectivity of the GR resulting in many nodes signalling through it to affect others. Thus, removal of this intermediary node results in a loss of signalling between model constituents.

In addition to the changes from ambivalent to no effect, numerous changes to and from other dependencies were observed across the numerous knockout scenarios. Across all knockout scenarios for the GEB052 model a total of 1249 dependency alterations was observed, which is reflective of the significant number of relationship changes that occur when network elements are lost. Even if changes from ambivalent factors to no effects are not considered (as there is no net change in positive or negative regulation) 323 predictions of dependency alterations (to or from activators or inhibitors) were seen. Although these all may exert physiological effects when translated from *in silico* to *in vivo*, it is anticipated that strong activators or strong inhibitors are the dependencies most likely to show an effect. Therefore there is a necessary focus on changes to or from strong inhibitors to strong activators, as has been performed previously for interactome modelling [19, 22].

For example, removal of the GR (which mimics GR mutation *in vivo*), resulted in the emergence of one strong inhibitor and one additional strong activator when compared to the wild type model (Table 2). In the unperturbed model DAP3 was ambivalent towards cell death whereas in the absence of the GR it became strong activator. In addition, STAT5B in the wild type model was ambivalent towards cell death whereas removal of the GR led to the dependency changing to strong inhibition. These predictions if confirmed by literature searches and laboratory-based experiments may have important clinical implications.

To assess the accuracy of the model published literature was surveyed to investigate whether the model predictions in dependency alterations have been previously observed in experimental research. The predictions that could not be verified by literature searching, were marked as a “Potentially Novel Prediction” [18] and the results are detailed in Table 3.

**Table 3:** Potentially novel predictions from dependency alterations.

Node Deleted	Node A	Node B	Wild-Type Dependency	KO Dependency	Verification (PubMed ID)	Consistent with Model Prediction?
GR	DAP3	CELL-DEATH	Ambivalent	Strong Activator	N/A	Potentially Novel Prediction
GR	STAT5B	CELL-DEATH	Ambivalent	Strong Inhibitor	N/A	Potentially Novel Prediction
HDAC1	DAXX	DAXX	Ambivalent	Strong Activator	N/A	Potentially Novel Prediction
HDAC1	DAXX	SUMO	Ambivalent	Strong Activator	N/A	Potentially Novel Prediction
HDAC1	SUMO	SUMO	Ambivalent	Strong Activator	N/A	Potentially Novel Prediction
HDAC1	SUMO	DAXX	Weak Activator	Strong Activator	N/A	Potentially Novel Prediction
HSP90	PRKDC	NCOA6	Weak Activator	Strong Activator	N/A	Potentially Novel Prediction
HSP90	NCOA6	NCOA6	Ambivalent	Strong Activator	N/A	Potentially Novel Prediction
HSP90	NCOA6	PRKDC	Ambivalent	Strong Activator	N/A	Potentially Novel Prediction
HSP90	PRKDC	PRKDC	Ambivalent	Strong Activator	N/A	Potentially Novel Prediction

Literature validation requires that the KO node, Node A and Node B are all mentioned; for instance, for row four of the above table, the paper would have to mention HDAC1 silencing or inhibition, which leads to DAXX activating SUMO. Otherwise, effects could be non-specific and not wholly consistent with model prediction. During the initial literature validation papers mentioning all three nodes could not be found.

However, some preliminary evidence has been gathered. The model predicted that in the absence of HSP90, PRKDC would be strongly activated by NCOA6 and by itself (likely via feedback loops). Corroborating this to some extent is one report which investigated the relationship between HSP90 and PRKDC (catalytic subunit of DNA-PK) and found that the use of the HSP90 inhibitor geldanamycin markedly enhanced TRAIL-induced DNA-PK [26]. However, this result was complicated as the same paper also showed that DNA-PK is a client of HSP90, which was required for full DNA-PK activation [26]. Thus, although the effector node (i.e. Node A) is not mentioned, it is promising that the overall outcome may correlate with model prediction.

Similar to the above, the model predicted that SUMO expression would be significantly higher following the loss of HDAC1. It has been shown that HDAC inhibition increases sumoylation in general, however the effect in one instance was mediated primarily through HDAC2 [27]. In addition, it has been demonstrated that HDAC1 inhibits sumoylation of a target protein therefore loss of HDAC1 would increase its sumoylation and thus the abundance of SUMO protein [28]. Again, this is consistent with model predictions, however the effector Node A (in this case DAXX or SUMO) has not been mentioned in this report.

Some model predictions were incorrect. The model predicted that loss of HDAC1 would lead to increased expression of DAXX; however, research has shown the opposite, with HDAC inhibitors leading to a decreased expression of DAXX [29]. But again, this paper does not specifically mention DAXX or SUMO as the effector node, so it is only a preliminary assessment of model accuracy.

### Genome-wide model analysis

**Fig 7:**
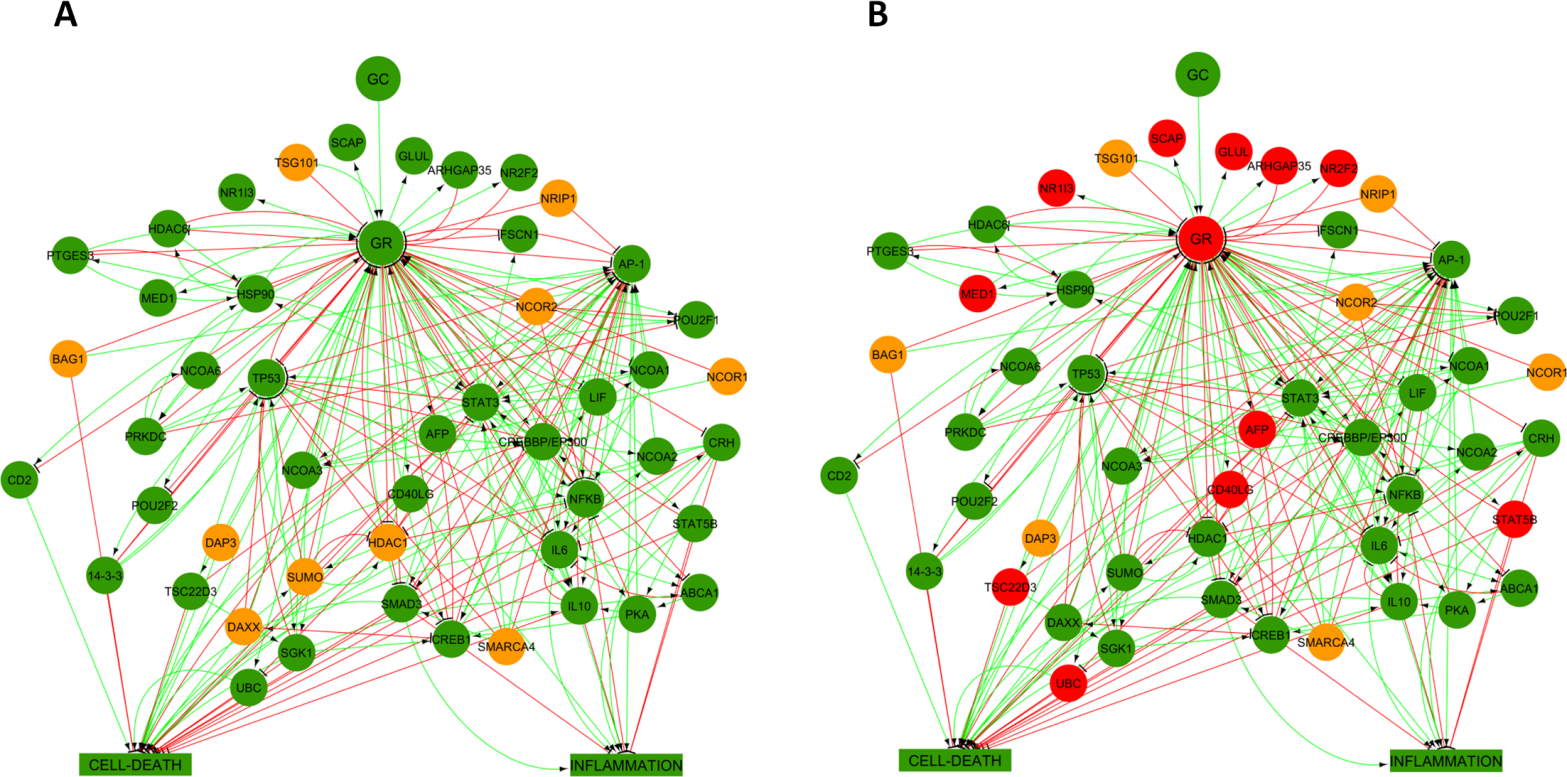
**Visualisation of LSSA results from glucocorticoid-sensitive (A) and glucocorticoid-resistant (B) simulations.** Nodes are coloured based on LSSA results: green indicates the node’s LSSA result was 1; orange indicates the node’s LSSA result was NaN and red indicates the node’s LSSA result was 0.

Although analysis of individual relationships via dependency matrices may provide insight into altered signalling, logical steady state analysis (LSSA) assesses the entirety of the model under different scenarios. The basal state for all model nodes is undetermined (NaN). Given a set of input values (i.e. GC = 1 for a glucocorticoid-sensitive simulation, or GC = 1, GR = 0 for a glucocorticoid-resistant simulation) LSSA will proceed to calculate the state (1/ON, NaN/undetermined or 0/OFF) of every downstream node within the model. Lastly, two LSSA scenarios may be compared to generate an *E*_*mod*_ value for each node which predicts the overall state change of the node between the two scenarios (1 = upregulated, 0 = no change, -1 = downregulated). Fig 7 provides a visual representation of the LSSA results for both the glucocorticoid-sensitive and glucocorticoid-resistant simulations, whilst Table 4 below summarises the LSSA results as well as the *E*_*mod*_ value for each node.

**Table 4:** LSSA results for glucocorticoid-sensitive and glucocorticoid-resistant simulations.

Node	GC-Sensitive (GC = 1) Simulation	GC-Resistant (GC = 1, GR = 0) Simulation	*E*_*mod*_
**14-3-3**	1	1	0
**ABCA1**	1	1	0
**AFP**	1	0	-1
**AP-1**	1	1	0
**ARHGAP35**	1	0	-1
**BAG1**	NaN	NaN	0
**CD2**	1	1	0
**CD40LG**	1	0	-1
**CELL-DEATH**	1	1	0
**CREB1**	1	1	0
**CREBBP/EP300**	1	1	0
**CRH**	1	1	0
**DAP3**	NaN	NaN	0
**DAXX**	NaN	1	1
**FSCN1**	1	1	0
**GC**	1	1	0
**GLUL**	1	0	-1
**GR**	1	0	-1
**HDAC1**	NaN	1	1
**HDAC6**	1	1	0
**HSP90**	1	1	0
**IL10**	1	1	0
**IL6**	1	1	0
**INFLAMMATION**	1	1	0
**LIF**	1	1	0
**MED1**	1	0	-1
**NCOA1**	1	1	0
**NCOA2**	1	1	0
**NCOA3**	1	1	0
**NCOA6**	1	1	0
**NCOR1**	NaN	NaN	0
**NCOR2**	NaN	NaN	0
**NFKB**	1	1	0
**NR1I3**	1	0	-1
**NR2F2**	1	0	-1
**NRIP1**	NaN	NaN	0
**PKA**	1	1	0
**POU2F1**	1	1	0
**POU2F2**	1	1	0
**PRKDC**	1	1	0
**PTGES3**	1	1	0
**SCAP**	1	0	-1
**SGK1**	1	1	0
**SMAD3**	1	1	0
**SMARCA4**	NaN	NaN	0
**STAT3**	1	1	0
**STAT5B**	1	0	-1
**SUMO**	NaN	1	1
**TP53**	1	1	0
**TSC22D3**	1	0	-1
**TSG101**	NaN	NaN	0
**UBC**	1	0	-1
**% ON**	80.8	63.5	
**% OFF**	0	23.1	
**% Determined**	80.8	86.6	
**% Undetermined**	19.2	13.4	

More determined (ON or OFF) nodes were seen in the glucocorticoid-resistant simulation, however this is balanced by the fact that a significantly higher number of nodes (23.1%) were OFF in the glucocorticoid-resistant simulation, which may reflect a loss of overall functionality within the network. The *E*_*mod*_ values (upregulated, no change, or downregulated) for nodes are summarised in Table 5.

**Table 5:** Node state comparison from glucocorticoid-sensitive to glucocorticoid-resistant simulations. Upregulated and downregulated refer to the fact that the node is more or less active in the glucocorticoid-resistant simulation than in the glucocorticoid-sensitive simulation.

Upregulated (3)	Unchanged (37)	Downregulated (12)
DAXX, HDAC1, SUMO	14-3-3, ABCA1, AP-1, BAG1, CREBBP/EP300, CD2, CELL-DEATH, CREB1, CRH, DAP3, FSCN1, GC, HDAC6, HSP90, IL10, IL6, INFLAMMATION, LIF, NCOA1, NCOA2, NCOA3, NCOA6, NCOR1, NCOR2, NFKB, NRIP1, PTGES3, TP53, PKA, POU2F1, POU2F2, PRKDC, SGK1, SMAD3, SMARCA4, STAT3, TSG101	AFP, NR1I3, CD40LG, GLUL, GR, ARHGAP35, MED1, NR2F2, SCAP, STAT5B, TSC22D3, UBC

Model predictions may be verified by literature searching in terms of experimental identification of upregulation or downregulation between glucocorticoid-sensitive and glucocorticoid-resistant cells. For instance, it has been shown that GLUL is downregulated in glucocorticoid-resistant cells [30]. However, a more practical approach to validating model LSSA predictions is the use of microarray data, detailed below.

Microarray data from glucocorticoid-sensitive and glucocorticoid-resistant cells were obtained from the Gene Expression Omnibus database and six comparisons were performed (as detailed in the Methods). The *E*_*mod*_ values obtained were compared to *E*_*exp*_ values created via comparison of a glucocorticoid-sensitive and glucocorticoid-resistant microarray. Comparison of these values gives the number of correct, small error and large error predictions within the model. The S1 Text file contains tables that show the *E*_*mod*_ and *E*_*exp*_ values in addition to their comparison for each microarray validation performed. Table 6 below summarises the overall number of correct/small error/large error predictions across all comparisons.

**Table 6:** Summary of prediction rates across all LSSA microarray validations.

Comparison	Correct (%)	Small Error (%)	Large Error (%)	P-Value of Correct Predictions
**1**	58.3	41.7	0.0	0.00022
**2**	54.2	43.8	2.1	0.00144
**3**	60.4	37.5	2.1	0.0000758621
**4**	58.3	39.6	2.1	0.00022
**5**	54.2	41.7	4.2	0.00144
**6**	54.2	45.8	0.0	0.00144
**AVERAGE**	56.6	41.7	1.8	0.000679172

As shown in Table 6, the GEB052 model generated accurate predictions across all scenarios (ranging from 54.2% to 60.4%, with an average of 56.6%). Given there are three possible outcomes (correct, small error and large error) a random model would achieve an expected correct prediction rate of 33.3%. The correct predictions from the six comparisons when compared to what a random model would achieve leads to a p-value <0.01, providing further evidence of the predictive capacity and potential of the GEB052 model.

### Preliminary clinical validation of the GEB052 model based on LSSA data

**Fig 8:**
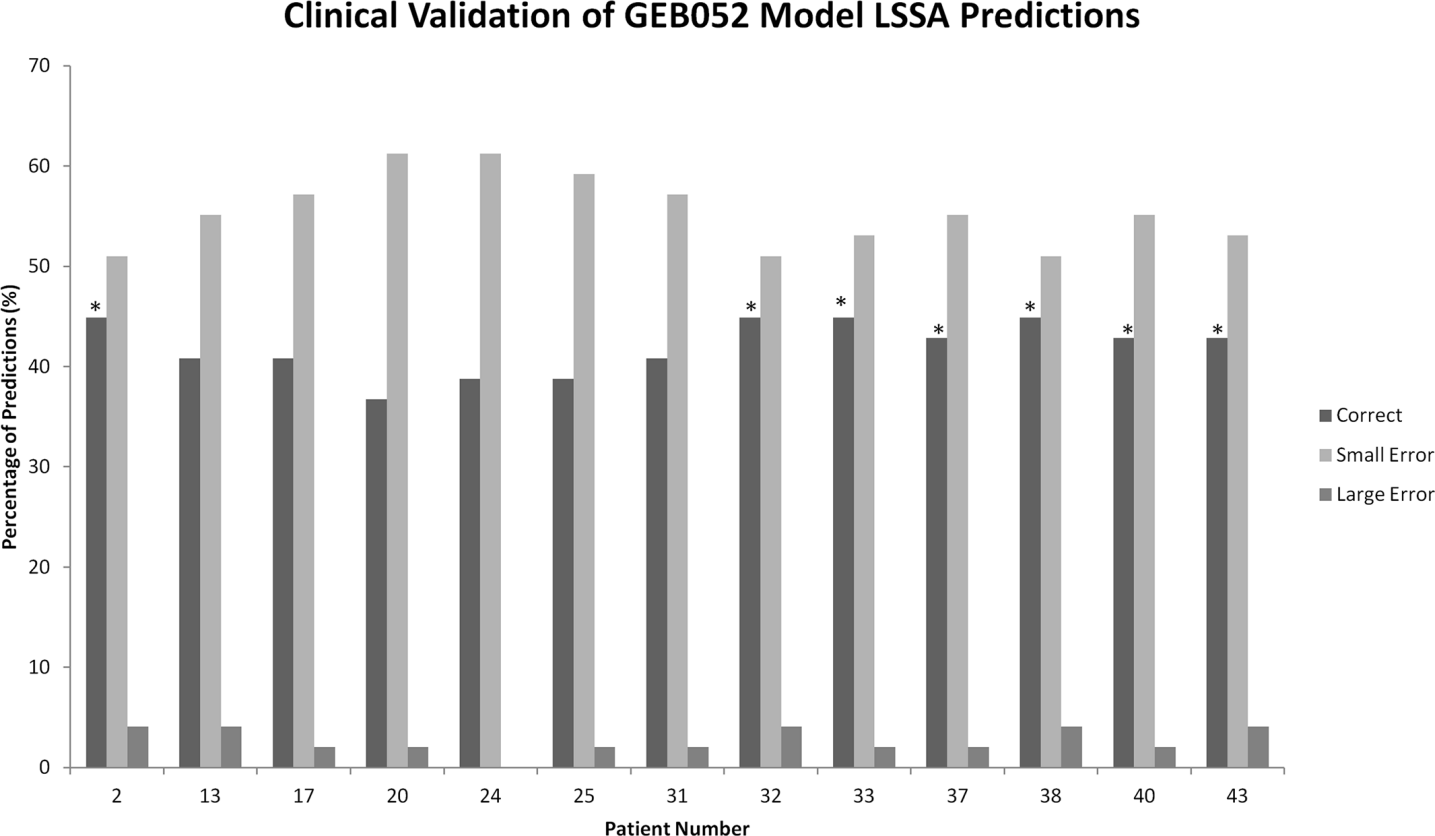
Clinical validation of GEB052 model via comparison of LSSA data to patient-based microarrays. The “Patient Number” on the x-axis refers to the patient number used in the original study [31] that these patients were taken from. An asterisk (*) indicates that the p-value of correct predictions for that patient was statistically significant at p<0.05.

In addition to the cell-based microarray data described above, the GEB052 model has been assessed as a predictive clinical tool (based on LSSA results) using microarray data from thirteen leukaemia patients (see the Methods section). The output of this result is shown in Fig 8, and the model’s LSSA results perform less well for analysis of individual patient data as an average correct prediction rate of 42% was observed, with 55% small error and 3% large error. Thus, although 55% of predictions were small error, the fact that large errors are still less than 5% is a promising indicator of the potential of the model. The fixed-state nature of LSSA (having only three discrete values) is a limitation on the analytical output which may partially explain this outcome, and thus a more quantitative analysis was taken next.

### Semi-quantitative model analysis via application of the signal transduction score flow algorithm

The GEB052 model has also undergone analysis using a semi-quantitative signal transduction score flow algorithm (STSFA) that superimposes ChIP-seq and/or microarray data onto a model to analyse the network with numerical data. The same comparisons for genome-wide validation was again utilised here (see Methods). The S1 Text file contains tables that show the calculation and output for each individual STSFA analysis and a summary is provided in Table 7.

**Table 7:** Prediction rates for the GEB052 model under STSFA analysis.

Comparison	Correct (%)	Small Error (%)	Large Error (%)	P-Value of Correct Predictions
**1**	82.6	17.4	0.0	7.53687×10^−12^
**2**	83.0	17.0	0.0	3.02763×10^−12^
**3**	87.2	12.8	0.0	2.58456×10^−14^
**4**	72.3	25.5	2.1	4.33425×10^−8^
**5**	74.5	23.4	2.1	8.04931×10^−9^
**6**	80.9	17.0	2.1	2.62395×10^−11^
**AVERAGE**	80.1	18.9	1.0	8.57144×10^−9^

**Fig 9:**
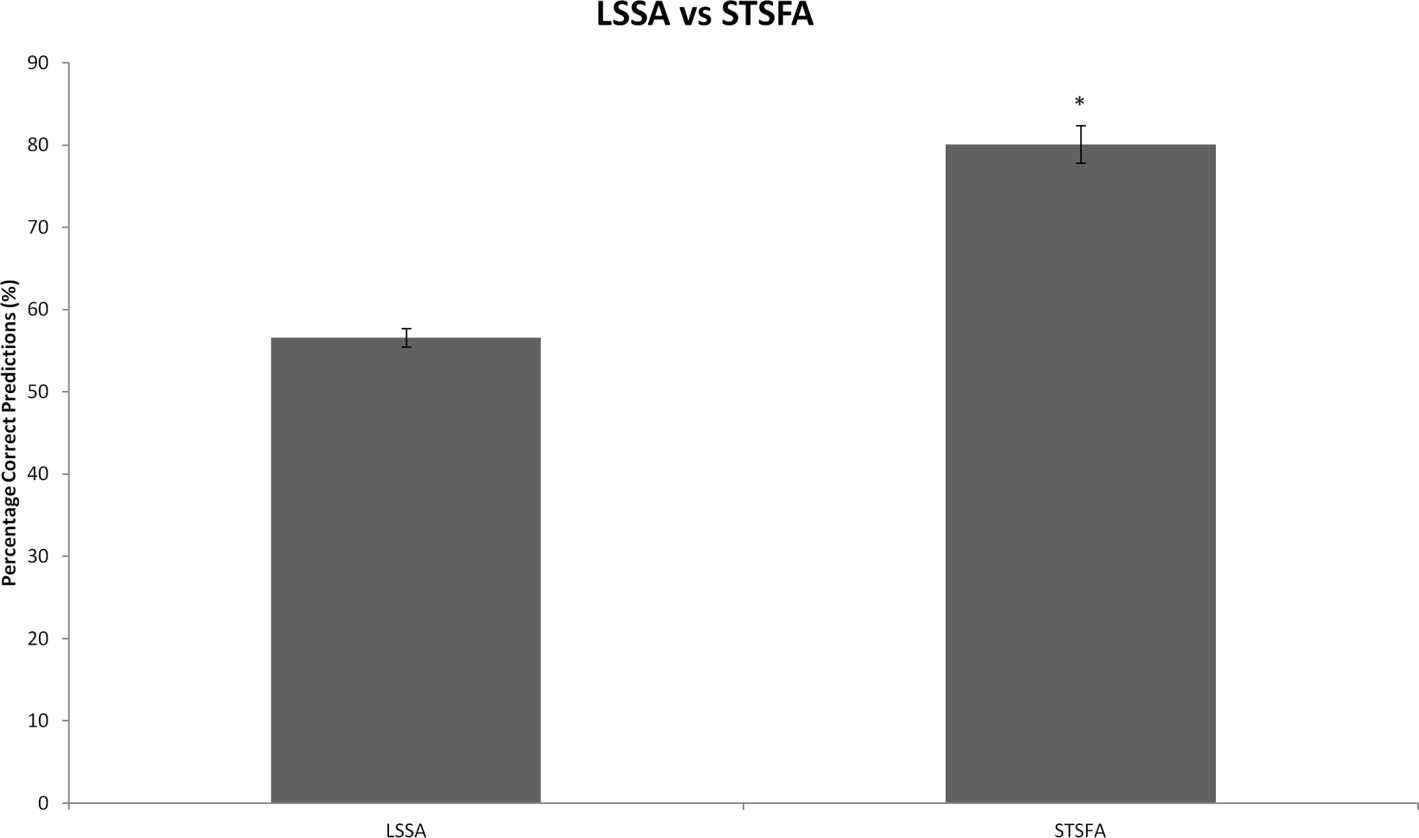
Correct predictions of LSSA against STSFA. Data represents the average correct prediction percentages +/- SEM. An asterisk (*) indicates p<0.05 as assessed by an unpaired t-test.

As shown in Table 7, STSFA analysis achieved significantly higher correct prediction rates than for discrete LSSA predictions (compare to Table 6). An average of 80.1% correct predictions was observed, with an average of 18.9% small error and 1.0% large error (and three out of six simulations exhibiting no large errors). The correct prediction rates for LSSA against STSFA have been graphed and compared via an unpaired t-test (Fig 9), which shows the enhanced predictive power that the semi-quantitative STSFA analysis offers. Due to this, assessment of the clinical potential of the GEB052 model with STSFA analysis was performed, as detailed below.

### Clinical predictive power of the GEB052 model under STSFA analysis

**Fig 10:**
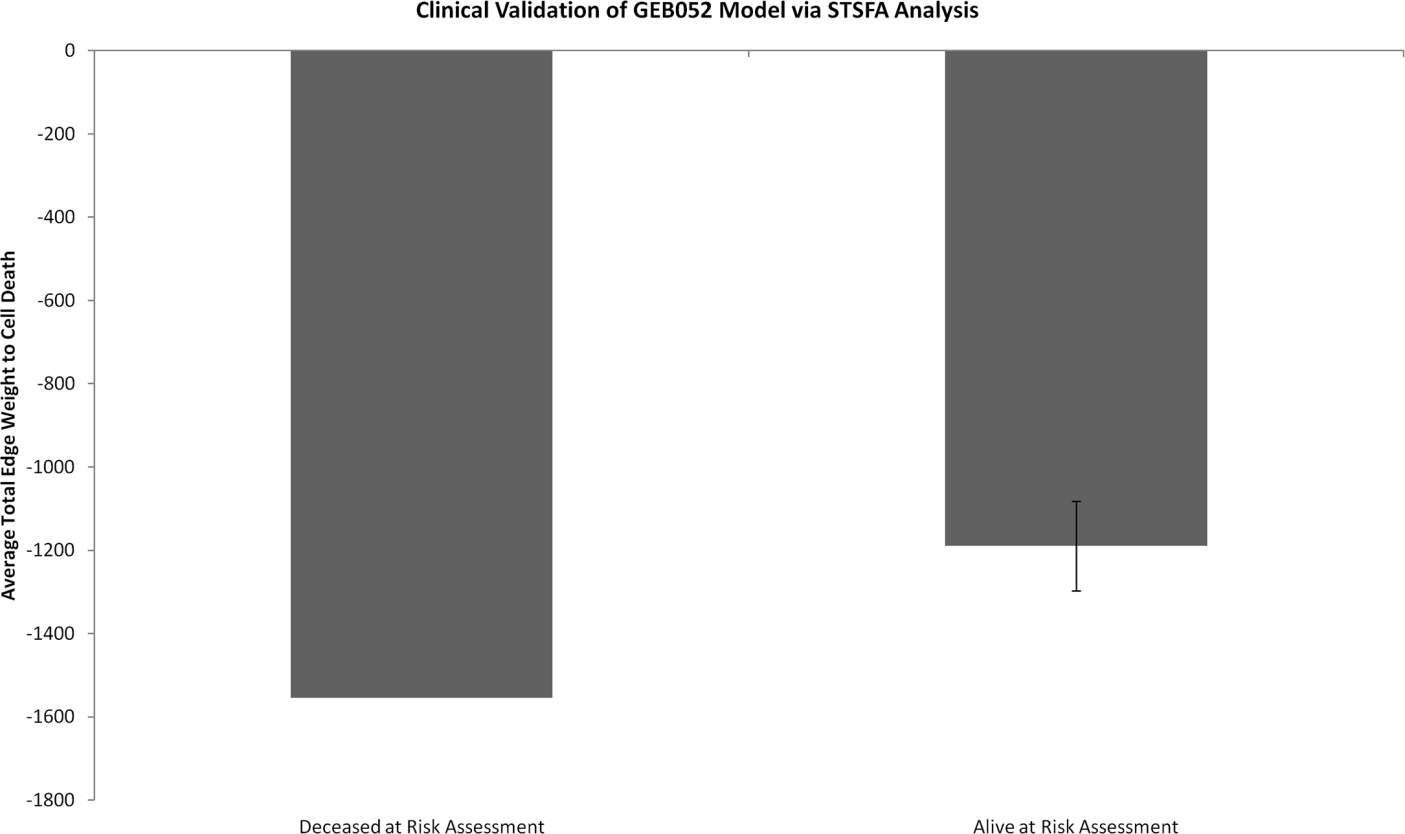
Clinical validation of the GEB052 model under STSFA analysis. The x-axis shows patient groups (Deceased at Risk Assessment, n = 1, Alive at Risk Assessment, n = 12) and the average for each group of the total edge weights targeting cell death +/- SEM are shown on the y-axis. Patient data taken from Schmidt and colleagues [31]and the GEO database.

Using microarray data from thirteen leukaemia patients (see Methods), the GEB052 model was analysed with the STSFA and the relative activation/inhibition of cell death was calculated for each patient. Patients were divided into two groups (twelve patients alive at risk assessment or one deceased at risk assessment) and the average +/- SEM (Fig 10). GEB052 model predictions indicated that the patient who died before risk assessment would have cell death more negatively regulated than those who were alive at risk assessment. Given that glucocorticoids are a chemotherapeutic drug for leukaemia, the cell death node in the model translates to death of the cancer cells *in vivo*. Thus, the model predicted that the patient who died before risk assessment would have cell death more negatively regulated; meaning that more cancer cells survive, in turn suggesting a worse prognosis. Thus, these preliminary model predictions correlate with clinical outcomes for the patients.

## Discussion

The widespread therapeutic use of glucocorticoids for many different diseases leads to a need to identify causes of therapy failure and glucocorticoid resistance. Systems biology offers the possibility of integrating the detailed knowledge of GR signalling to generate models that can be used to gain insight into how the network functions following a loss of GR function.

Computational research methodologies have been previously applied to GR research using approaches such as virtual ligand screening [32], development of models to quantitatively model specific signalling events [17] or the creation of models that aim to simulate glucocorticoid receptor control of both directly-regulated and indirectly-regulated genes [33]. Each of these approaches have provided insight to GR signalling, however to date a Boolean interactome model of the glucocorticoid receptor has not been developed. To generate the GEB052 model, the STRING database was to provide a basis for the interactions to be included. Following the generation of all the model links between the proteins interacting with the GR, model outputs in the form of cell death and inflammation were added via Gene Ontology and manual curation.

Interactome modelling has previously been applied to cancer research, such as the development of the original PKT206 p53 interactome and the later expanded PMH260 interactome [18, 19]. These models, in addition to the application of the STSFA to the PKT206 model [22] all showed good predictive ratios and thus a similar approach was undertaken here to model GR signalling.

The GEB052 model consists of 52 nodes connected by 241 logical interactions, and has 64 two-step feedback loops within the model. Comparatively, the PKT206 model had only 30 two-step feedback loops, whilst the expanded PMH260 model had only 34 feedback loops [18, 19]. The identification of 64 two-step feedback loops within the GEB052 model is particularly interesting as the GEB052 model is significantly smaller than PKT206 or PMH260, containing only 52 nodes compared to 206 or 260. Thus, despite the model being significantly smaller the network appears to be much more integrated and interconnected, which may explain the fact that the majority of nodes were unchanged between the sensitive and resistant LSSA simulation results, as well as potentially explaining the fewer number of changes to strong activators or inhibitors following dependency matrix generation in KO scenarios; the PKT206 model identified 63 changes to/from strong activators or inhibitors, whilst only ten were seen in the GEB052 model. This is due to the fact that feedback loops have been previously identified as important in the robustness of a network [18]. Although this initial analysis has focussed only on strong activators or inhibitors, 323 changes to or from activators or inhibitors were identified across all knockout scenarios, and thus examination of these dependency alterations would represent a source of future work.

The validation of model LSSA results through cell-based microarray data indicated an average of 56.60% correct predictions, 41.67% small error and 1.74% large error. The PKT206 interactome model displayed a correct prediction range from 52–71% [18]. The correct prediction range for the GEB052 model was lower (54.17% to 60.42%), less large errors were seen in the GEB052 model validation; two out of six comparisons yielded no large errors, whilst the other four led to a large error range of only 2.08% to 4.17%. It is important to note that the expanded PMH260 interactome displayed less large errors than the original PKT206 model, and therefore model expansion represents an additional source of future work for the GEB052 model.

Validation of model LSSA results with patient microarray data yielded lower correct prediction rates (an average of 42%). However, it is still promising that large error predictions comprised the minority of prediction outcomes, suggesting some potential of the model. Furthermore, working on the assumption that a random model would achieve a correct prediction rate of 33.3%, then a 42% correct average from thirteen sets of data is statistically significantly higher (p<0.0001).This lower correct prediction rate could be attributed to a variety of factors, including the relatively small size of the model, as well as the complexity of translating findings from a simulation of a small gene regulatory network to a whole organism level. Additionally, as specified in the introduction, effects of GCs are very cell-type specific; the GR may differentially modulate genes depending on the type of tissue. Thus, an additional way to further develop the GR model is through the incorporation of tissue-specific interactions and the development of cell-specific forms of the model, although the relevant literature required for this is currently incomplete.

Consistent with previous research [22] the STSFA demonstrated a statistically significantly higher level of correct prediction rates (80.1% for STSFA compared to 56.6% for LSSA). Curiously, large error predictions for STSFA appeared only in microarray data from B-ALL and not T-ALL, which indicates that the model may predict T-ALL to a better standard than B-ALL. Furthermore, the enhanced predictive power of STSFA (likely due to its semi-quantitative nature) provided a justification for its use in clinical assessment. By using microarray data from thirteen leukaemia patients (taken before chemotherapy treatment, and thus analytical outcomes represent true predictions) Fig 10 was generated. The fact that the model predicted that the patient who died before risk assessment would have cell death being more inhibited (equating to death of the cancer cells) is promising, as it provides a potential link between model prediction and clinical outcome. However, this analysis is admittedly preliminary due to the fact that there are a small number of patients (13) and one group had only one patient, whilst the other had twelve. Thus, although promising, further assessment with a larger patient cohort is needed.

Ultimately, the GEB052 model construction, validation, and clinical assessment represent a proof of principle of the applicability of this approach to glucocorticoid receptor research. The GEB052 model under Boolean analysis provides good predictive ratios for cell-based microarray data, and application of the semi-quantitative STSFA to the model demonstrated even higher correct predictive rates. Lastly, the use of the GEB052 model under STSFA analysis has also shown promise at the clinical level using microarray data from thirteen leukaemia patients. Key points for future development include model expansion and incorporation of tissue-specific reactions. In addition, it is recognised that there are multiple isoforms of the GR, each of which can have different effects on downstream nodes, and in fact interactions between different GR isoforms can be a determinant of its activity [34]. Thus, it may also be useful to develop models of different GR isoforms to better represent physiological occurrences. Regardless of future directions, the GEB052 model represents a promising starting point and potential clinical tool given its predictive ratios and the correlation of its STSFA output with patient clinical outcomes. Application of individual patient data to the model could thus be a stepping stone towards personalised therapy.

## Methods

### Extraction and manual curation of STRING data

STRING (Search Tool for the Retrieval of Interacting Genes/Proteins, v9.1 at the time of curation) was used as the database of known and predicted protein interactions [24]. Extraction and filtering of data was performed in a similar manner as described previously [18]. The “protein.actions.v9.1.txt.gz” file was downloaded from STRING and all high confidence (≥ 0.7) interactions for the glucocorticoid receptor were then extracted. TSC22D3 (GILZ, glucocorticoid-induced leucine zipper) and EP300 were also included due to their known importance in GR signalling or similarity to CREBBP respectively.

Manual curation of STRING data was then undertaken via extensive literature searches of the two putative interacting proteins. STRING includes various interaction modes such as “activation”, “inhibition” and “binding”. In all cases, manual curation was undertaken to confirm STRING records, and also to uncover any functional relationships between the two genes that were not included in STRING. Manual curation was essential as the nature of the STRING database (such as being based on text mining) results in the possibility of incorrect interactions being retained in the database. It has previously been shown that multiple types of errors can occur such as incorrect gene name recognition [18].

After manual curation of all the interactions with the GR (the “primary layer”), all high-confidence interactions for the proteins that were shown to interact with GR were extracted. This list was then filtered to retain interactions only between the proteins which appeared in the primary layer. Additional curation was then undertaken in order to verify STRING data (the “second layer”), and thus after this a closed two-layer model was produced. All curations of predicted interactions were double-curated to improve model reliability.

### Connection to model outputs through the Gene Ontology (GO) consortium

The GO database [35] was used to provide biological outputs for the model. Following completion of the second layer, GO terms/annotations were collected for each node of the network, pooled together and ranked by the most common, and then the most common terms related to biological outputs were chosen. This lead to several groups of GO terms: cell death; inflammation; immune response; metabolism; development; cell growth and proliferation. For this first version of the model only cell death and inflammation were chosen as outputs due to their relevance in glucocorticoid therapy. For all model links to outputs, manual double-curation was again undertaken to verify interactions.

### Cytoscape

Model visualisation was undertaken through the use of Cytoscape, an open-source software for data visualisation [36]. Curated interaction records were imported into the program and visualised after adjusting parameters. Application of the STSFA (below) was also conducted in Cytoscape.

### Model analysis through CellNetAnalyzer

CellNetAnalyzer (CNA, v2017.1c) is a MATLAB toolbox which allows for the analysis of gene-regulatory models based on the topology of the interaction network. Interactions between nodes of the network are represented through hypergraphs which can allow for interaction combinations such as OR functions or the use of AND functions, both of which allow for more accurate representation of true biological reactions (such as several proteins forming a complex to activate or inhibit a target) [23]. CNA was used to construct Boolean signal flow networks. At present, the model presented herein does not contain AND reactions; in cases where interactions converge to the same node the combination follows OR logic by default. Inhibitory reactions are represented by a NOT modifier. These logics have been described in detail by Klamt and colleagues [23].

Several types of analysis are available through CNA, such as the generation of an interaction matrix (which summarises the participation of each node in every reaction), logical steady state analysis (LSSA) and the generation of dependency matrices. By defining (i.e. ON or OFF) the state of nodes (particularly input nodes) of the model, LSSA will calculate the steady state of network nodes downstream of the input based on the interactions within the model. Three node states are possible under LSSA: 1 (ON), 0 (OFF) or NaN (undetermined). A node may be assigned NaN if multiple states are possible; this may be caused by input conditions being insufficient to determine all node states, or through feedback loops leading to multiple steady states and oscillatory behaviour [18, 37].

The second main approach used in CellNetAnalyzer is the generation of dependency matrices. A dependency matrix provides a visual and numerical representation of the overall relationships between the nodes of the network, taking into account all of the interactions within the model (thus allowing indirect functional relationships to be considered). Six different types of dependencies are possible based on the relationship between nodes in the interaction:

1. A has no effect on B if there are no positive or negative paths from A to B
2. A is a strong activator of B if there are positive paths from A to B, and no negative paths from A to B. It is also required that there are no negative feedback loops within these positive paths.
3. A is a weak activator of B if there are positive paths from A to B, no negative paths from A to B, and there are negative feedback loops within these positive paths.
4. A is a strong inhibitor of B if there are negative paths from A to B and no positive paths from A to B. It is also required that there are no negative feedback loops within these negative paths.
5. A is a weak inhibitor of B if there are negative paths from A to B, no positive paths from A to B, and there are negative feedback loops within these negative paths.
6. A is ambivalent towards B if there are both positive and negative paths from A to B.

Comparison of the dependency matrices from the full model to a modified (i.e. KO model) can unveil modified relationships and signalling. Because model KOs simulate *in vivo* loss-of-function mutations, these matrix comparisons provide predictions for how cells will behave. These predictions may then be verified in the laboratory to assess model predictive power and model accuracy [18, 19].

### Comparison of LSSA result scenarios

Comparisons between two sets of LSSA results (such as a GC-sensitive scenario against a GC-resistant scenario) were also carried out as previously described [18], which allows for the assessment of node upregulation or downregulation between two scenarios.

In brief, LSSA calculates the state (inactivated (0), undetermined (NaN) or activated (1)) of nodes within the network following a set of input value(s). For Scenario 1 (i.e. a GC-sensitive simulation), node *i* state was defined as *S(i)*_*1*_ which has a value of NaN, 0, or 1. For Scenario 2 (i.e. a GC-resistant simulation), node *i* state was defined as *S(i)*_*2*_, which may also have a value of NaN, 0, or 1. Lastly the value *E*_*mod*_ was used to define the predicted change in node state from Scenario 1 to Scenario 2, where 1 means the node is upregulated, -1 means the node is downregulated and 0 means the node state is unchanged:

$$\begin{array}{l} E_{mod}=\text{-}1\;\mathrm{if}\;S{\left(i\right)}_{1}=1\;\mathrm{and}\;S{\left(i\right)}_{2}=0\\ E_{mod}=\text{-}1\;\mathrm{if}\;S{\left(i\right)}_{1}=1\;\mathrm{and}\;S{\left(i\right)}_{2}=\mathrm{NaN}\\ E_{mod}=\text{-}1\;\mathrm{if}\;S{\left(i\right)}_{1}=\mathrm{NaN}\;\mathrm{and}\;S{\left(i\right)}_{2}=0 \end{array}$$

$$\begin{array}{l} E_{mod}=0\;\mathrm{if}\;S{\left(i\right)}_{1}=1\;\mathrm{and}\;S{\left(i\right)}_{2}=1\\ E_{mod}=0\;\mathrm{if}\;S{\left(i\right)}_{1}=0\;\mathrm{and}\;S{\left(i\right)}_{2}=0\\ E_{mod}=0\;\mathrm{if}\;S{\left(i\right)}_{1}=\mathrm{NaN}\;\mathrm{and}\;S{\left(i\right)}_{2}=\mathrm{NaN} \end{array}$$

$$\begin{array}{l} E_{mod}=1\;\mathrm{if}\;S{\left(i\right)}_{1}=0\;\mathrm{and}\;S{\left(i\right)}_{2}=1\\ E_{mod}=1\;\mathrm{if}\;S{\left(i\right)}_{1}=\mathrm{NaN}\;\mathrm{and}\;S{\left(i\right)}_{2}=1\\ E_{mod}=1\;\mathrm{if}\;S{\left(i\right)}_{1}=0\;\mathrm{and}\;S{\left(i\right)}_{2}=\mathrm{NaN} \end{array}$$

### Model validation through microarray data

Consistent with previous publications [18, 19] the predictions generated by the GEB052 model were assessed against microarray data from GC-resistant and GC-sensitive cells. Twelve microarrays were obtained from the Gene Expression Omnibus (GEO) database, and the following six comparisons were utilised as shown in Table 8.

**Table 8:** Comparisons for genome-wide model validation.

Comparison	GC-Sensitive Array	GC-Resistant Array
Comparison 1	T-ALL (C7H2 Cells), 24 Hours Dexamethasone Treatment (GEO ID GSM60544)	T-ALL (C1 Cells), 24 Hours Dexamethasone Treatment (GEO ID GSM60562)
Comparison 2	T-ALL (C7H2 Cells), 6 Hours Dexamethasone Treatment (GEO ID GSM60543)	T-ALL (C1 Cells), 6 Hours Dexamethasone Treatment (GEO ID GSM60561)
Comparison 3	T-ALL (C7H2 Cells), 6 Hours 0.1% Ethanol Treatment (GEO ID GSM60542)	T-ALL (C1 Cells), 6 Hours 0.1% Ethanol Treatment (GEO ID GSM60560)
Comparison 4	B-ALL (PreB 697 Cells), 24 Hours Dexamethasone Treatment (GEO ID GSM60547)	B-ALL (PreB 697 R4G4 Cells), 24 Hours Dexamethasone Treatment (GEO ID GSM60586)
Comparison 5	B-ALL (PreB 697 Cells), 6 Hours Dexamethasone Treatment (GEO ID GSM60546)	B-ALL (PreB 697 R4G4 Cells), 6 Hours Dexamethasone Treatment (GEO ID GSM60583)
Comparison 6	B-ALL (PreB 697 Cells), 6 Hours 0.1% Ethanol Treatment (GEO ID GSM60545)	B-ALL (PreB 697 R4G4 Cells), 6 Hours 0.1% Ethanol Treatment (GEO ID GSM60581)

Differential expression analysis was performed using the dynamic threshold method used by Tian and colleagues and Hussain and colleagues [18, 19] and the expression change (*E*_*exp*_) value was calculated, where 1 equates to upregulation, 0 to no change, and -1 to downregulation. Using the GC-resistant array as the target scenario, and the GC-sensitive array as the source scenario, fold changes for all microarray probe IDs were generated between the target and source scenarios. The Log_10_ for all fold changes was calculated, and a dynamic threshold based on the average Log_10_ fold change + the standard deviation (upper limit) and the average Log_10_ fold change–the standard deviation (lower limit).

For each gene present in the model, the median value for all probe IDs relevant to the gene was calculated for both the source and target scenario, in addition to the fold change of the median values. For model nodes which represented the combination of multiple genes (i.e. the AP-1 node which represents FOS and JUN) the median value for all probe IDs for all of its constituents was used. Log_10_ values of these fold changes were calculated and compared to the dynamic threshold; if higher than the upper limit, the gene was determined as upregulated (*E*_*exp*_ = 1), whilst if the value was lower than the lower limit the gene was determined as downregulated (*E*_*exp*_ = -1) and if its value lay between the lower and upper limits then the gene was determined as unchanged (*E*_*exp*_ = 0).

To evaluate model performance, the absolute value of *E*_*mod*_*−E*_*exp*_ was calculated, which could take three possible values: 0 (no difference between *E*_*mod*_ and *E*_*exp*_; model prediction was correct); 1 (small difference between *E*_*mod*_ and *E*_*exp*_; small error prediction) and 2 (large difference between *E*_*mod*_ and *E*_*exp*_; large error prediction meaning that the model predicted the opposite of what occurred in cells).

### Assessment of model predictions (LSSA) using individual patient data

Table 9 shows the microarray data used for clinical validation of LSSA results.

**Table 9:** Microarray data used for clinical validation of LSSA data. Data taken from Schmidt and colleagues [31].

Patient Number	Gender	Age (Years)	Clustering	Status at Risk Assessment?	GEO ID
2	M	8.5	T-ALL	Alive	GSM51710
13	M	5.9	Not assigned	Alive	GSM51677
17	F	14.7	Hyperploidy	Deceased	GSM51680
20	M	5	T-ALL	Alive	GSM51704
24	M	2.6	Not assigned	Alive	GSM51674
25	F	10.3	T-ALL	Alive	GSM51707
31	F	17.2	Hyperploidy	Alive	GSM51683
32	F	3.7	TEL-AML	Alive	GSM51686
33	M	2.5	Hyperploidy	Alive	GSM51689
37	F	15.1	Not assigned	Alive	GSM51692
38	M	3.2	TEL-AML	Alive	GSM51695
40	M	17.3	Not assigned	Alive	GSM51698
43	F	1.6	TEL-AML	Alive	GSM51701

To compare model LSSA results with clinical data from patients, thirteen microarrays (detailed in Table 9) from leukaemia patients (taken following treatment with prednisolone) were obtained from the GEO database [31]. For each individual patient, Log_**10**_ RMA values for all probe IDs were calculated and a dynamic threshold based on the average +/- standard deviation was generated. The median Log_**10**_ values for all the probe IDs for genes within the model were then compared to the threshold: if the value was higher than the upper limit, the gene was considered as upregulated; if the value was lower than the lower limit, the gene was considered as downregulated and if the value lay between the lower and upper limits then the gene was unchanged. These values were then compared to model LSSA results of a GC-sensitive simulation, where 1 is equivalent to upregulated, 0 to downregulated and NaN to unchanged.

### Application of the STSFA to GEB052

The STSFA plugin for Cytoscape [21] was used to apply the STSFA to the model. As with previous studies [19] Log_2_ RMA values were scaled up by a factor of 100 and superimposed onto the model using the pathway scoring application. A limitation of the STSFA is that it apparently cannot handle directly ambivalent relationships; that is, if Node A both directly activates and inhibits Node B, the STSFA cannot accurately handle this. To correct for this, all directly ambivalent relationships were removed prior to the application of the STSFA. Mathematically, this is not unreasonable as even if the direct ambivalent interactions were considered, the overall regulation would be zero as it would theoretically be positively and negatively affected by equal amounts. The same twelve microarray datasets listed in Table 8 were used for STSFA analysis.

STSFA results from the GC-sensitive and GC-resistant arrays were used to generate an *E*_*mod*_ value, whilst the *E*_*exp*_ values for each Comparison were the same as for the cell-based microarray genome-wide model validation. To generate the *E*_*mod*_ values, fold changes between the node scores of the resistant output and sensitive output were generated, followed by the Log_10_ of the fold changes. From the Log_10_ fold changes for each node a dynamic threshold based on the average +/- standard deviation was generated, and nodes were considered as upregulated if their score was higher than the upper limit, downregulated if their score was lower than the lower limit, and unchanged if the score lay between the two. These *E*_*mod*_ values were compared to the *E*_*exp*_ values to assess model accuracy in the same way as the cell-based microarray genome-wide model validation.

### Preliminary assessment of GEB052 as a predictive clinical tool (STSFA using patient data)

To assess the potential of the GEB052 model as a predictive clinical tool, microarray data from thirteen leukaemia patients (taken before patients were treated) was obtained from the GEO database, following its deposit after the original study that generated the data [31] (Table 10).

**Table 10:** Patient microarray data used for STSFA analysis. Data taken from Schmidt and colleagues [31].

Patient Number	Gender	Age (Years)	Clustering	Status at Risk Assessment?	GEO ID
2	M	8.5	T-ALL	Alive	GSM51712
13	M	5.9	Not assigned	Alive	GSM51679
17	F	14.7	Hyperploidy	Deceased	GSM51682
20	M	5	T-ALL	Alive	GSM51706
24	M	2.6	Not assigned	Alive	GSM51676
25	F	10.3	T-ALL	Alive	GSM51709
31	F	17.2	Hyperploidy	Alive	GSM51685
32	F	3.7	TEL-AML	Alive	GSM51688
33	M	2.5	Hyperploidy	Alive	GSM51691
37	F	15.1	Not assigned	Alive	GSM51694
38	M	3.2	TEL-AML	Alive	GSM51697
40	M	17.3	Not assigned	Alive	GSM51700
43	F	1.6	TEL-AML	Alive	GSM51703

For each patient the STSFA was used to superimpose their microarray data onto the model. The STSFA assigns node score to every node within the model, in addition to calculating weights for each of the edges (indicating the “strength” of the regulation). Patients were split into two groups (alive at risk assessment or dead at risk assessment). The total incoming edge weights to cell death (one output of the GEB052 model) was calculated for each patient, and an average made for each group, in addition to calculating the SEM. This analysis thus correlated model predictions with clinical outcomes.

### P-Value calculation for correct predictions

To assess whether the correct prediction rates of the model were statistically significant, the WolframAlpha computational knowledge engine (http://www.wolframalpha.com/) was used in conjunction with the search term “Probability of [X] success in [Y] trials, chance of success is [Z]”. In these cases [X] equates to the number of correct predictions, [Y] to the total number of predictions and [Z] to the chance of success (one in three, as there are three possible outcomes).

### Determination of incorrect nodes

To determine if any nodes were systematically incorrect across the comparisons shown in Table 8, the absolute values of *E*_*mod*_*−E*_*exp*_ were totalled for each node and all comparisons. As previously stated, the absolute value of *E*_*mod*_*−E*_*exp*_ can take three possible values: 0 (correct), 1 (small error) and 2 (large error). A threshold of four (indicating that the node had small errors in more than 50% of comparisons, or had two large errors) was chosen to determine a node as incorrect. The incorrect node determination for cell-based microarray validation is shown in the S1 Text file.

## Supporting information

S1 Text This additional file contains the supplementary tables relevant to this manuscript. These tables cover information such as the list of interactions within the GEB052 model, GEB052 model validation and STSFA analysis, and the determination of systematically incorrect nodes.
(DOCX)
