# Supplementary material for: Insight into glucocorticoid receptor signalling through interactome model analysis
Source: PLoS Comput Biol. 2017 Nov 6;13(11):e1005825. doi: 10.1371/journal.pcbi.1005825 (PMC5690696; doi:10.1371/journal.pcbi.1005825)
Supplement: S1 Text — These tables cover information such as the list of interactions within the GEB052 model, GEB052 model validation and STSFA analysis, and the determination of systematically incorrect nodes. (DOCX) [file pcbi.1005825.s001.docx]

**Supplementary Text (Supporting Information)**

**S1 Table:** Nodes consisting of more than one protein within the GEB052 model or nodes requiring additional explanation.

| **Node** | **Constituents/Explanation** |
| --- | --- |
| 14-3-3 | SFN/YWHAS, YWHAB, YWHAE, YWHAG, YWHAH, YWHAQ, and YWHAZ |
| AP-1 | FOS and JUN |
| NFKB | RELA and NFKB1 |
| CREBBP/EP300 | CREBBP and EP300 |
| SUMO | SUMO1, SUMO2, SUMO3 and SUMO4 |
| PKA | Contains only the interactions for PRKACG (gamma catalytic subunit of PKA) but is named as such to serve as an intermediary between CRH/AP-1 and ABCA1/IL10 |
| HSP90 | Contains only the interactions for HSP90AA1 (HSP90alpha) |

**S2 Table:** List of primary layer interactions within the GEB052 model.

| **Node 1** | **Activates (1) or Inhibits (0)** | **Node 2** | **PubMed ID Evidence** |
| --- | --- | --- | --- |
| 14-3-3 | 0 | GR | 12730237 |
| 14-3-3 | 1 | GR | 9079630, 11266503, 16338219 |
| AP-1 | 0 | GR | 8388998 |
| AP-1 | 1 | GR | 8388998 |
| ARHGAP35 | 0 | GR | 10385430, 20427664 , 1894621 |
| BAG1 | 0 | GR | 11101523, 9603979, 19595997. |
| CREB1 | 0 | GR | 1387550 |
| CREBBP/EP300 | 0 | GR | 8616895, 10528999 |
| CREBBP/EP300 | 1 | GR | 17884810, 9792627, 10528999 |
| DAP3 | 1 | GR | 10903152, 12099703 |
| DAXX | 0 | GR | 12595526, 17081986 |
| GC | 1 | GR | No PMID - Logical Input For Model |
| GR | 0 | ABCA1 | 16254209 |
| GR | 1 | ABCA1 | 17241464 |
| GR | 1 | AFP | 11549270 |
| GR | 0 | AP-1 | 2169352, 2169353, 9731701 |
| GR | 1 | AP-1 | 7583019 |
| GR | 1 | ARHGAP35 | 1894621 |
| GR | 0 | CD2 | 9144521 |
| GR | 1 | CD2 | 9172010 |
| GR | 1 | CD40LG | 11160161 |
| GR | 0 | CREB1 | 21804312 |
| GR | 1 | CREB1 | 21804312, 14668092 |
| GR | 1 | CREBBP/EP300 | 23125313 |
| GR | 0 | CRH | 22232675, 19177170 |
| GR | 0 | FSCN1 | 10026156 |
| GR | 1 | GLUL | 10453053, 14962810 |
| GR | 0 | HDAC1 | 16762839, 12727880 |
| GR | 0 | HDAC6 | 20538901 |
| GR | 1 | IL10 | 10940925, 16341266 |
| GR | 0 | IL6 | 22042221, 12114264, 11007957 |
| GR | 0 | LIF | 10080876, 8432990, 7579343, 9099902 |
| GR | 1 | MED1 | 17827210 |
| GR | 0 | NCOA1 | 11196413, 12039076 |
| GR | 0 | NFKB | 7823959, 8290595 |
| GR | 1 | NFKB | 9885901, 23693080 |
| GR | 1 | NR1I3 | 11093784, 12511605, 15557560 |
| GR | 1 | NR2F2 | 14739255 |
| GR | 1 | PKA | 16319314 |
| GR | 1 | POU2F1 | 9584182 |
| GR | 0 | POU2F2 | 1714379 |
| GR | 1 | POU2F2 | 9584182 |
| GR | 1 | SCAP | 15133039 |
| GR | 1 | SGK1 | 22590650, 17595317 |
| GR | 0 | SMAD3 | 10518526 |
| GR | 0 | STAT3 | 20881248 |
| GR | 1 | STAT3 | 14522952 |
| GR | 1 | STAT5B | 9973262, 15037546 |
| GR | 0 | TP53 | 11562347, 11080152 |
| GR | 1 | TSC22D3 | 9430225, 16239257 |
| GR | 1 | UBC | 17875808, 11872750, 10913373, |
| HDAC1 | 0 | GR | 15826950 |
| HDAC1 | 1 | GR | 15826950, 16762839 |
| HDAC6 | 0 | GR | 20018896 |
| HSP90 | 0 | GR | 2005120, 16087666 |
| HSP90 | 1 | GR | 2005120, 16087666 |
| IL10 | 1 | GR | 16341266 |
| LIF | 0 | GR | 15985451 |
| MED1 | 1 | GR | 17827210, 10508170, 16239257, 19630272 |
| NCOA1 | 1 | GR | 12569182, 16339206 |
| NCOA2 | 1 | GR | 19805480 |
| NCOA3 | 1 | GR | 11094166, 16179382 |
| NCOA6 | 1 | GR | 10567404 |
| NCOR1 | 0 | GR | 12011091, 12569182, 23428870 |
| NCOR2 | 0 | GR | 10598585 |
| NFKB | 0 | GR | 7823959, 8290595 |
| NR2F2 | 0 | GR | 14739255 |
| NRIP1 | 0 | GR | 10364267, 12773562 |
| POU2F1 | 1 | GR | 9584182 |
| PRKDC | 0 | GR | 9038175 |
| PTGES3 | 0 | GR | 17261597 |
| PTGES3 | 1 | GR | 17438133 |
| SGK1 | 1 | GR | 23650397 |
| SMAD3 | 1 | GR | 12753290 |
| SMARCA4 | 0 | GR | 17043312 |
| STAT3 | 1 | GR | 9388192, 12904256,20204302 |
| STAT5B | 0 | GR | 8878484 |
| SUMO | 0 | GR | 12193561 |
| SUMO | 1 | GR | 12193561 |
| TP53 | 0 | GR | 9215863 |
| TSG101 | 0 | GR | 10508170, 15033475 |
| TSG101 | 1 | GR | 15657031 |

**S3 Table:** List of second layer interactions in the GEB052 model.

| **Node 1** | **Activates (1) or Inhibits (0)** | **Node 2** | **PubMed ID Evidence** |
| --- | --- | --- | --- |
| 14-3-3 | 0 | TP53 | 18339856 |
| 14-3-3 | 1 | TP53 | 9620776, 14517281 |
| ABCA1 | 0 | IL6 | 19783654, 17079792 |
| ABCA1 | 1 | PKA | 23055522 |
| AP-1 | 1 | CREB1 | 11976688, 1827203 |
| AP-1 | 1 | IL10 | 16569682, 22634314, 15067049 |
| AP-1 | 1 | IL6 | 8453101, 15158360, 20833374 |
| AP-1 | 0 | NFKB | 12181357 |
| AP-1 | 1 | NFKB | 9468519, 8404856 |
| AP-1 | 0 | SMAD3 | 10903323, 10871633, 17660955 |
| AP-1 | 1 | SMAD3 | 16730810, 11134003 |
| AP-1 | 1 | STAT3 | 11356008 |
| AP-1 | 0 | TP53 | 10072388, 11136975 |
| BAG1 | 1 | AP-1 | 11329370, 20516211 |
| CREB1 | 0 | AP-1 | 1840296, 2140898 |
| CREB1 | 1 | AP-1 | 12432566, 9770464, 16151051, 2140898 |
| CREB1 | 1 | IL10 | 19564345, 16920714, 18263767 |
| CREBBP/EP300 | 0 | AP-1 | 11689449 |
| CREBBP/EP300 | 1 | AP-1 | 7588605, 21937452, 9388250, 10327051, 8545107 |
| CREBBP/EP300 | 1 | CREB1 | 13678586 |
| CREBBP/EP300 | 0 | HDAC1 | 16762839 |
| CREBBP/EP300 | 1 | NCOA1 | 15688032 |
| CREBBP/EP300 | 1 | NCOA2 | 20448036, 9430642, 10899170 |
| CREBBP/EP300 | 0 | NFKB | 12419806 |
| CREBBP/EP300 | 1 | NFKB | 9890939, 9096323, 11739381 |
| CREBBP/EP300 | 1 | SMAD3 | 9679056 |
| CREBBP/EP300 | 1 | STAT3 | 15649887, 15653507 |
| CREBBP/EP300 | 0 | TP53 | 19805293 |
| CREBBP/EP300 | 1 | TP53 | 18485870, 9288775 |
| CRH | 0 | IL6 | 10922080, 11602623, 1731761 |
| CRH | 1 | IL6 | 1623564, 8246669 |
| CRH | 1 | PKA | 1663213 17895291, 11960782, 7783858, 8793851, 11325519 (N.B. Full pathway is CRH --> PKA --> AP-1) |
| DAXX | 0 | CREB1 | 22185778 |
| DAXX | 1 | SUMO | 17081986 |
| DAXX | 0 | TP53 | 15570294, 15364927 |
| DAXX | 1 | TP53 | 14557665 |
| HDAC1 | 0 | CREB1 | 12567184 |
| HDAC1 | 0 | CREBBP/EP300 | 14968110 |
| HDAC1 | 0 | NFKB | 11931769 |
| HDAC1 | 0 | SMAD3 | 16876108 |
| HDAC1 | 0 | STAT3 | 15653507, 18611949 |
| HDAC1 | 1 | SUMO | 18025037 |
| HDAC1 | 0 | TP53 | 12426395, 10777477 |
| HDAC1 | 0 | UBC | 19304753 |
| HDAC6 | 1 | HSP90 | 15916966, 19158084, 18591380 |
| HSP90 | 1 | HDAC6 | 21109931 |
| HSP90 | 1 | PRKDC | 22753480, 16263121 |
| HSP90 | 1 | PTGES3 | 15040786 |
| HSP90 | 1 | STAT3 | 12559950, 18339423, 12235142 |
| IL10 | 1 | ABCA1 | 16336952, 20354139 |
| IL10 | 0 | AP-1 | 9864163, 8709636, 20045008 |
| IL10 | 0 | IL6 | 12017175 |
| IL10 | 1 | IL6 | 8703029 |
| IL10 | 1 | STAT3 | 8830676 |
| IL6 | 1 | ABCA1 | 21757719 |
| IL6 | 1 | AP-1 | 10465257, 9240403, 17018293, 8398910 |
| IL6 | 1 | CRH | 1846105, 2845968, 10465257 |
| IL6 | 1 | IL10 | 23349310 |
| IL6 | 1 | LIF | 12151548 |
| IL6 | 1 | NCOA2 | 19240160 |
| IL6 | 1 | STAT3 | 17065510, 18160665 |
| IL6 | 0 | TP53 | 15930285, 1852210 |
| LIF | 1 | AP-1 | 9711940, 9545305, 8621626, 1628710, 8917449, 2144331 |
| LIF | 1 | IL6 | 11160255 |
| LIF | 1 | STAT3 | 9813052 |
| NCOA1 | 1 | AP-1 | 9642216, 10847592, 18511550, 16860316 |
| NCOA1 | 1 | CREBBP/EP300 | 15688032 |
| NCOA1 | 1 | NCOA3 | 20685850 |
| NCOA1 | 1 | NFKB | 9556555 |
| NCOA1 | 1 | STAT3 | 11773079 |
| NCOA2 | 1 | AP-1 | 18511550 |
| NCOA2 | 1 | CREBBP/EP300 | 15731352, 20448036 |
| NCOA3 | 1 | CREBBP/EP300 | 10866661 |
| NCOA3 | 1 | NCOA1 | 20685850 |
| NCOA6 | 1 | PRKDC | 12519782 |
| NCOR1 | 1 | NCOA3 | 12089344 |
| NCOR2 | 1 | NCOA3 | 20392877 |
| NCOR2 | 0 | NFKB | 10777532 |
| NCOR2 | 0 | POU2F1 | 11134019 |
| NFKB | 1 | AP-1 | 8404856 |
| NRIP1 | 0 | AP-1 | 12554755, 10379892 |
| PKA | 1 | AP-1 | 1663213 17895291, 11960782, 7783858, 8793851, 11325519 (N.B. Full pathway is CRH --> PKA --> AP-1) |
| PKA | 1 | CREB1 | 15337521 |
| PKA | 1 | IL10 | 23055522 |
| PKA | 0 | NFKB | 15642694 |
| PRKDC | 1 | HSP90 | 22270370, 19021771 |
| PRKDC | 1 | NCOA6 | 12519782 |
| PRKDC | 0 | POU2F1 | 14612514 |
| PRKDC | 1 | POU2F1 | 17213819 |
| PRKDC | 1 | TP53 | 9363941 |
| PTGES3 | 0 | HSP90 | 11812147 |
| PTGES3 | 1 | HSP90 | 9148915, 11060043 |
| SGK1 | 1 | CREB1 | 15733869 |
| SGK1 | 0 | TP53 | 19756449 |
| SMAD3 | 0 | AP-1 | 14752027 |
| SMAD3 | 1 | AP-1 | 21829441, 9125213 |
| SMARCA4 | 0 | AP-1 | 12372840, 10082538 |
| SMARCA4 | 1 | AP-1 | 11053448 |
| SMARCA4 | 1 | SMAD3 | 21900401, 18003620 |
| SMARCA4 | 1 | STAT3 | 21785422 |
| SMARCA4 | 0 | TP53 | 19448667, 18822392 |
| STAT3 | 1 | AP-1 | 16205632, 9271408, 11319221, 7568080, 10490649, 20463008, 12600988, 19404962 (mutual activation due to widespread synergy) |
| STAT3 | 1 | FSCN1 | 21937440 |
| STAT3 | 1 | HSP90 | 9461509, 23228483 |
| STAT3 | 1 | IL10 | 19234181 |
| STAT3 | 1 | IL6 | 19751774, 19284588, 18160665, 21122157 |
| STAT3 | 0 | TP53 | 16107692, 22303479 |
| SUMO | 0 | AP-1 | 16055710, 10788439, 23396363 |
| SUMO | 1 | DAXX | 17081986 |
| SUMO | 0 | HDAC1 | 11960997 |
| SUMO | 1 | HDAC1 | 24068740 |
| SUMO | 0 | TP53 | 21900752 |
| SUMO | 1 | TP53 | 17012228 |
| TP53 | 1 | 14-3-3 | 14517281, 17546054 |
| TP53 | 1 | CREBBP/EP300 | 9194564 |
| TP53 | 0 | IL6 | 21092249, 11830554 |
| TP53 | 1 | SGK1 | 19756449, 8647846 |
| TSC22D3 | 0 | AP-1 | 11397794, 12391160 |
| TSC22D3 | 1 | SGK1 | 20947508 |

**S4 Table:** List of GEB052 model links to cell death.

| **Node 1** | **Activates (1) or Inhibits (0)** | **Node 2** | **PubMed ID Evidence** |
| --- | --- | --- | --- |
| 14-3-3 | 0 | CELL-DEATH | 12426317, 11222372, 24626062, 22562251 |
| AP-1 | 1 | CELL-DEATH | 10080190 |
| AP-1 | 0 | CELL-DEATH | 9141200 |
| BAG1 | 0 | CELL-DEATH | 11257006 |
| CD2 | 1 | CELL-DEATH | 9270771 |
| CD40LG | 0 | CELL-DEATH | 12697681 |
| CD40LG | 1 | CELL-DEATH | 12885753 |
| CRH | 0 | CELL-DEATH | 23380766 |
| CRH | 1 | CELL-DEATH | 23686762, 22494987, 11790788, 22763913. |
| DAP3 | 1 | CELL-DEATH | 20563667, 17135360 |
| DAXX | 1 | CELL-DEATH | 1598338 |
| GR | 1 | CELL-DEATH | 15940303, 21530661, 12039857 |
| IL10 | 0 | CELL-DEATH | 8312229 |
| IL10 | 1 | CELL-DEATH | 9184696 |
| IL6 | 0 | CELL-DEATH | 7595060, 11751424 |
| IL6 | 1 | CELL-DEATH | 9949178 |
| NFKB | 0 | CELL-DEATH | 10747850, 10849002 |
| NFKB | 1 | CELL-DEATH | 10747850 |
| SGK1 | 0 | CELL-DEATH | 17571248 |
| SMAD3 | 0 | CELL-DEATH | 14517210 |
| SMAD3 | 1 | CELL-DEATH | 15107418, 11839804 |
| STAT3 | 0 | CELL-DEATH | 23807222 |
| STAT3 | 1 | CELL-DEATH | 21336304 |
| STAT5B | 0 | CELL-DEATH | 21826656 |
| TP53 | 1 | CELL-DEATH | 19879762 |
| TSC22D3 | 0 | CELL-DEATH | 9430225 |
| UBC | 0 | CELL-DEATH | 17491588 |
| UBC | 1 | CELL-DEATH | 15033975, 15620210 |

**S5 Table:** List of GEB052 model links to inflammation.

| **Node 1** | **Activates (1) or Inhibits (0)** | **Node 2** | **PMID** |
| --- | --- | --- | --- |
| AP-1 | 1 | INFLAMMATION | 23163821 |
| CD40LG | 1 | INFLAMMATION | 9468137 |
| CRH | 0 | INFLAMMATION | 17117478 |
| CRH | 1 | INFLAMMATION | 17117478 |
| IL10 | 0 | INFLAMMATION | 10443688, 12452830, 14971032 |
| IL6 | 1 | INFLAMMATION | 2199284, 10443688, 25031389 |
| NFKB | 0 | INFLAMMATION | 18270204, 20457564 |
| NFKB | 1 | INFLAMMATION | 18029230, 23776175, 20457564 |
| SMAD3 | 0 | INFLAMMATION | 14752027 |
| SMAD3 | 1 | INFLAMMATION | 20231525, 15253712 |
| STAT5B | 0 | INFLAMMATION | 24412367 |
| STAT5B | 1 | INFLAMMATION | 17148664 |

For S6 Table – S17 Table below, in the *E_mod_* and *E_exp_* columns, “0” means the node/protein is unchanged between the glucocorticoid-sensitive and glucocorticoid-resistant scenario, “-1” means the node/protein is downregulated in the glucocorticoid-resistant scenario and “1” means the node/protein is upregulated in the glucocorticoid-resistant scenario. In the ABS (*E_mod_-E_exp_*) column, “0” means the prediction was correct, “1” means there was a small error prediction and “2” means there was a large error prediction.

**S6 Table:** GEB052 model validation via microarray data (Comparison 1).

| **Comparison 1** | ***E_mod_*** | ***E_exp_*** | **ABS (*E_mod_-E_exp_*)** |
| --- | --- | --- | --- |
| **14-3-3.** | 0 | 0 | 0 |
| **ABCA1** | 0 | 0 | 0 |
| **AFP** | -1 | 0 | 1 |
| **AP-1** | 0 | -1 | 1 |
| **ARHGAP35** | -1 | 0 | 1 |
| **BAG1** | 0 | 0 | 0 |
| **CD2** | 0 | 0 | 0 |
| **CD40LG** | -1 | 0 | 1 |
| **CREB1** | 0 | 0 | 0 |
| **CREBBP/EP300** | 0 | 0 | 0 |
| **CRH** | 0 | 0 | 0 |
| **DAP3** | 0 | 0 | 0 |
| **DAXX** | 1 | 0 | 1 |
| **FSCN1** | 0 | 1 | 1 |
| **GLUL** | -1 | -1 | 0 |
| **HDAC1** | 1 | 0 | 1 |
| **HDAC6** | 0 | 0 | 0 |
| **HSP90** | 0 | 0 | 0 |
| **IL10** | 0 | -1 | 1 |
| **IL6** | 0 | 0 | 0 |
| **LIF** | 0 | 0 | 0 |
| **MED1** | -1 | 0 | 1 |
| **NCOA1** | 0 | -1 | 1 |
| **NCOA2** | 0 | 0 | 0 |
| **NCOA3** | 0 | 0 | 0 |
| **NCOA6** | 0 | 0 | 0 |
| **NCOR1** | 0 | 0 | 0 |
| **NCOR2** | 0 | 0 | 0 |
| **NFKB** | 0 | -1 | 1 |
| **NR1I3** | -1 | 0 | 1 |
| **NR2F2** | -1 | 0 | 1 |
| **NRIP1** | 0 | 0 | 0 |
| **PKA** | 0 | 0 | 0 |
| **POU2F1** | 0 | 0 | 0 |
| **POU2F2** | 0 | 0 | 0 |
| **PRKDC** | 0 | 0 | 0 |
| **PTGES3** | 0 | 0 | 0 |
| **SCAP** | -1 | 0 | 1 |
| **SGK1** | 0 | -1 | 1 |
| **SMAD3** | 0 | -1 | 1 |
| **SMARCA4** | 0 | 1 | 1 |
| **STAT3** | 0 | 0 | 0 |
| **STAT5B** | -1 | 0 | 1 |
| **SUMO** | 1 | 0 | 1 |
| **TP53** | 0 | 0 | 0 |
| **TSC22D3** | -1 | -1 | 0 |
| **TSG101** | 0 | 0 | 0 |
| **UBC** | -1 | 0 | 1 |
|  | | | |
| **Correct** | 28 | 58.3% |  |
| **Small Error** | 20 | 41.7% |  |
| **Large Error** | 0 | 0.0% |  |

**S7 Table:** GEB052 model validation via microarray data (Comparison 2).

| **Comparison 2** | ***E_mod_*** | ***E_exp_*** | **ABS (*E_mod_-E_exp_*)** |
| --- | --- | --- | --- |
| **14-3-3.** | 0 | 0 | 0 |
| **ABCA1** | 0 | -1 | 1 |
| **AFP** | -1 | 0 | 1 |
| **AP-1** | 0 | -1 | 1 |
| **ARHGAP35** | -1 | 0 | 1 |
| **BAG1** | 0 | 0 | 0 |
| **CD2** | 0 | 0 | 0 |
| **CD40LG** | -1 | 0 | 1 |
| **CREB1** | 0 | 1 | 1 |
| **CREBBP/EP300** | 0 | 0 | 0 |
| **CRH** | 0 | 0 | 0 |
| **DAP3** | 0 | 0 | 0 |
| **DAXX** | 1 | 0 | 1 |
| **FSCN1** | 0 | 1 | 1 |
| **GLUL** | -1 | -1 | 0 |
| **HDAC1** | 1 | 0 | 1 |
| **HDAC6** | 0 | 0 | 0 |
| **HSP90** | 0 | 0 | 0 |
| **IL10** | 0 | -1 | 1 |
| **IL6** | 0 | 0 | 0 |
| **LIF** | 0 | 0 | 0 |
| **MED1** | -1 | 0 | 1 |
| **NCOA1** | 0 | 0 | 0 |
| **NCOA2** | 0 | 0 | 0 |
| **NCOA3** | 0 | 1 | 1 |
| **NCOA6** | 0 | 0 | 0 |
| **NCOR1** | 0 | 0 | 0 |
| **NCOR2** | 0 | 0 | 0 |
| **NFKB** | 0 | 0 | 0 |
| **NR1I3** | -1 | 0 | 1 |
| **NR2F2** | -1 | 0 | 1 |
| **NRIP1** | 0 | 1 | 1 |
| **PKA** | 0 | 0 | 0 |
| **POU2F1** | 0 | 0 | 0 |
| **POU2F2** | 0 | 1 | 1 |
| **PRKDC** | 0 | 0 | 0 |
| **PTGES3** | 0 | 0 | 0 |
| **SCAP** | -1 | 0 | 1 |
| **SGK1** | 0 | 0 | 0 |
| **SMAD3** | 0 | -1 | 1 |
| **SMARCA4** | 0 | 0 | 0 |
| **STAT3** | 0 | 0 | 0 |
| **STAT5B** | -1 | 0 | 1 |
| **SUMO** | 1 | -1 | 2 |
| **TP53** | 0 | 0 | 0 |
| **TSC22D3** | -1 | 0 | 1 |
| **TSG101** | 0 | 0 | 0 |
| **UBC** | -1 | 0 | 1 |
|  | | | |
| **Correct** | 26 | 54.2% |  |
| **Small Error** | 21 | 43.8% |  |
| **Large Error** | 1 | 2.1% |  |

**S8 Table:** GEB052 model validation via microarray data (Comparison 3).

| **Comparison 3** | ***E_mod_*** | ***E_exp_*** | **ABS (*E_mod_-E_exp_*)** |
| --- | --- | --- | --- |
| **14-3-3.** | 0 | 0 | 0 |
| **ABCA1** | 0 | 0 | 0 |
| **AFP** | -1 | 0 | 1 |
| **AP-1** | 0 | -1 | 1 |
| **ARHGAP35** | -1 | 0 | 1 |
| **BAG1** | 0 | 0 | 0 |
| **CD2** | 0 | 0 | 0 |
| **CD40LG** | -1 | 0 | 1 |
| **CREB1** | 0 | 0 | 0 |
| **CREBBP/EP300** | 0 | 0 | 0 |
| **CRH** | 0 | 0 | 0 |
| **DAP3** | 0 | 0 | 0 |
| **DAXX** | 1 | 0 | 1 |
| **FSCN1** | 0 | 1 | 1 |
| **GLUL** | -1 | -1 | 0 |
| **HDAC1** | 1 | 0 | 1 |
| **HDAC6** | 0 | 0 | 0 |
| **HSP90** | 0 | 0 | 0 |
| **IL10** | 0 | 0 | 0 |
| **IL6** | 0 | 0 | 0 |
| **LIF** | 0 | 0 | 0 |
| **MED1** | -1 | 0 | 1 |
| **NCOA1** | 0 | 0 | 0 |
| **NCOA2** | 0 | 0 | 0 |
| **NCOA3** | 0 | 1 | 1 |
| **NCOA6** | 0 | 0 | 0 |
| **NCOR1** | 0 | 0 | 0 |
| **NCOR2** | 0 | 0 | 0 |
| **NFKB** | 0 | 0 | 0 |
| **NR1I3** | -1 | 0 | 1 |
| **NR2F2** | -1 | 0 | 1 |
| **NRIP1** | 0 | 1 | 1 |
| **PKA** | 0 | 0 | 0 |
| **POU2F1** | 0 | 0 | 0 |
| **POU2F2** | 0 | 0 | 0 |
| **PRKDC** | 0 | 0 | 0 |
| **PTGES3** | 0 | 0 | 0 |
| **SCAP** | -1 | 0 | 1 |
| **SGK1** | 0 | 0 | 0 |
| **SMAD3** | 0 | -1 | 1 |
| **SMARCA4** | 0 | 1 | 1 |
| **STAT3** | 0 | 0 | 0 |
| **STAT5B** | -1 | 0 | 1 |
| **SUMO** | 1 | -1 | 2 |
| **TP53** | 0 | 0 | 0 |
| **TSC22D3** | -1 | 0 | 1 |
| **TSG101** | 0 | 0 | 0 |
| **UBC** | -1 | 0 | 1 |
|  | | | |
| **Correct** | 29 | 60.4% |  |
| **Small Error** | 18 | 37.5% |  |
| **Large Error** | 1 | 2.1% |  |

**S9 Table:** GEB052 model validation via microarray data (Comparison 4).

| **Comparison 4** | ***E_mod_*** | ***E_exp_*** | **ABS (*E_mod_-E_exp_*)** |
| --- | --- | --- | --- |
| **14-3-3.** | 0 | 0 | 0 |
| **ABCA1** | 0 | 1 | 1 |
| **AFP** | -1 | -1 | 0 |
| **AP-1** | 0 | 0 | 0 |
| **ARHGAP35** | -1 | 0 | 1 |
| **BAG1** | 0 | 0 | 0 |
| **CD2** | 0 | 0 | 0 |
| **CD40LG** | -1 | 0 | 1 |
| **CREB1** | 0 | 0 | 0 |
| **CREBBP/EP300** | 0 | 0 | 0 |
| **CRH** | 0 | 0 | 0 |
| **DAP3** | 0 | 1 | 1 |
| **DAXX** | 1 | 0 | 1 |
| **FSCN1** | 0 | -1 | 1 |
| **GLUL** | -1 | -1 | 0 |
| **HDAC1** | 1 | 0 | 1 |
| **HDAC6** | 0 | 0 | 0 |
| **HSP90** | 0 | 0 | 0 |
| **IL10** | 0 | 0 | 0 |
| **IL6** | 0 | 0 | 0 |
| **LIF** | 0 | -1 | 1 |
| **MED1** | -1 | 1 | 2 |
| **NCOA1** | 0 | -1 | 1 |
| **NCOA2** | 0 | 0 | 0 |
| **NCOA3** | 0 | 0 | 0 |
| **NCOA6** | 0 | 0 | 0 |
| **NCOR1** | 0 | 0 | 0 |
| **NCOR2** | 0 | 0 | 0 |
| **NFKB** | 0 | -1 | 1 |
| **NR1I3** | -1 | 0 | 1 |
| **NR2F2** | -1 | 0 | 1 |
| **NRIP1** | 0 | 0 | 0 |
| **PKA** | 0 | 0 | 0 |
| **POU2F1** | 0 | 0 | 0 |
| **POU2F2** | 0 | -1 | 1 |
| **PRKDC** | 0 | 0 | 0 |
| **PTGES3** | 0 | 0 | 0 |
| **SCAP** | -1 | -1 | 0 |
| **SGK1** | 0 | 1 | 1 |
| **SMAD3** | 0 | 0 | 0 |
| **SMARCA4** | 0 | 0 | 0 |
| **STAT3** | 0 | -1 | 1 |
| **STAT5B** | -1 | 0 | 1 |
| **SUMO** | 1 | 0 | 1 |
| **TP53** | 0 | -1 | 1 |
| **TSC22D3** | -1 | -1 | 0 |
| **TSG101** | 0 | 0 | 0 |
| **UBC** | -1 | 0 | 1 |
|  | | | |
| **Correct** | 28 | 58.3% |  |
| **Small Error** | 19 | 39.6% |  |
| **Large Error** | 1 | 2.1% |  |

**S10 Table:** GEB052 model validation via microarray data (Comparison 5).

| **Comparison 5** | ***E_mod_*** | ***E_exp_*** | **ABS (*E_mod_-E_exp_*)** |
| --- | --- | --- | --- |
| **14-3-3.** | 0 | 0 | 0 |
| **ABCA1** | 0 | 1 | 1 |
| **AFP** | -1 | 1 | 2 |
| **AP-1** | 0 | 0 | 0 |
| **ARHGAP35** | -1 | 0 | 1 |
| **BAG1** | 0 | 0 | 0 |
| **CD2** | 0 | 0 | 0 |
| **CD40LG** | -1 | 0 | 1 |
| **CREB1** | 0 | 0 | 0 |
| **CREBBP/EP300** | 0 | 0 | 0 |
| **CRH** | 0 | 0 | 0 |
| **DAP3** | 0 | 1 | 1 |
| **DAXX** | 1 | -1 | 2 |
| **FSCN1** | 0 | -1 | 1 |
| **GLUL** | -1 | -1 | 0 |
| **HDAC1** | 1 | 0 | 1 |
| **HDAC6** | 0 | 0 | 0 |
| **HSP90** | 0 | 0 | 0 |
| **IL10** | 0 | 0 | 0 |
| **IL6** | 0 | 0 | 0 |
| **LIF** | 0 | 0 | 0 |
| **MED1** | -1 | 0 | 1 |
| **NCOA1** | 0 | 0 | 0 |
| **NCOA2** | 0 | 0 | 0 |
| **NCOA3** | 0 | 0 | 0 |
| **NCOA6** | 0 | 0 | 0 |
| **NCOR1** | 0 | -1 | 1 |
| **NCOR2** | 0 | -1 | 1 |
| **NFKB** | 0 | -1 | 1 |
| **NR1I3** | -1 | 0 | 1 |
| **NR2F2** | -1 | 0 | 1 |
| **NRIP1** | 0 | -1 | 1 |
| **PKA** | 0 | 0 | 0 |
| **POU2F1** | 0 | 0 | 0 |
| **POU2F2** | 0 | 0 | 0 |
| **PRKDC** | 0 | -1 | 1 |
| **PTGES3** | 0 | 0 | 0 |
| **SCAP** | -1 | -1 | 0 |
| **SGK1** | 0 | 0 | 0 |
| **SMAD3** | 0 | 0 | 0 |
| **SMARCA4** | 0 | -1 | 1 |
| **STAT3** | 0 | 0 | 0 |
| **STAT5B** | -1 | 0 | 1 |
| **SUMO** | 1 | 0 | 1 |
| **TP53** | 0 | -1 | 1 |
| **TSC22D3** | -1 | 0 | 1 |
| **TSG101** | 0 | 0 | 0 |
| **UBC** | -1 | 0 | 1 |
|  | | | |
| **Correct** | 26 | 54.2% |  |
| **Small Error** | 20 | 41.7% |  |
| **Large Error** | 2 | 4.2% |  |

**S11 Table:** GEB052 model validation via microarray data (Comparison 6).

| **Comparison 6** | ***E_mod_*** | ***E_exp_*** | **ABS (*E_mod_-E_exp_*)** |
| --- | --- | --- | --- |
| **14-3-3.** | 0 | 0 | 0 |
| **ABCA1** | 0 | 1 | 1 |
| **AFP** | -1 | 0 | 1 |
| **AP-1** | 0 | 0 | 0 |
| **ARHGAP35** | -1 | 0 | 1 |
| **BAG1** | 0 | 0 | 0 |
| **CD2** | 0 | 0 | 0 |
| **CD40LG** | -1 | 0 | 1 |
| **CREB1** | 0 | 0 | 0 |
| **CREBBP/EP300** | 0 | 0 | 0 |
| **CRH** | 0 | 0 | 0 |
| **DAP3** | 0 | 1 | 1 |
| **DAXX** | 1 | 0 | 1 |
| **FSCN1** | 0 | -1 | 1 |
| **GLUL** | -1 | 0 | 1 |
| **HDAC1** | 1 | 0 | 1 |
| **HDAC6** | 0 | 0 | 0 |
| **HSP90** | 0 | 0 | 0 |
| **IL10** | 0 | 1 | 1 |
| **IL6** | 0 | 0 | 0 |
| **LIF** | 0 | 0 | 0 |
| **MED1** | -1 | 0 | 1 |
| **NCOA1** | 0 | 0 | 0 |
| **NCOA2** | 0 | 0 | 0 |
| **NCOA3** | 0 | 0 | 0 |
| **NCOA6** | 0 | 0 | 0 |
| **NCOR1** | 0 | -1 | 1 |
| **NCOR2** | 0 | 0 | 0 |
| **NFKB** | 0 | 0 | 0 |
| **NR1I3** | -1 | 0 | 1 |
| **NR2F2** | -1 | 0 | 1 |
| **NRIP1** | 0 | -1 | 1 |
| **PKA** | 0 | 0 | 0 |
| **POU2F1** | 0 | 0 | 0 |
| **POU2F2** | 0 | 0 | 0 |
| **PRKDC** | 0 | -1 | 1 |
| **PTGES3** | 0 | 0 | 0 |
| **SCAP** | -1 | -1 | 0 |
| **SGK1** | 0 | 0 | 0 |
| **SMAD3** | 0 | 0 | 0 |
| **SMARCA4** | 0 | -1 | 1 |
| **STAT3** | 0 | 0 | 0 |
| **STAT5B** | -1 | -1 | 0 |
| **SUMO** | 1 | 0 | 1 |
| **TP53** | 0 | -1 | 1 |
| **TSC22D3** | -1 | 0 | 1 |
| **TSG101** | 0 | 1 | 1 |
| **UBC** | -1 | 0 | 1 |
|  | | | |
| **Correct** | 26 | 54.2% |  |
| **Small Error** | 22 | 45.8% |  |
| **Large Error** | 0 | 0.0% |  |

**S12 Table:** Model validation by STSFA analysis (Comparison 1). TP53 is excluded from the accuracy analysis as its final score for the glucocorticoid-sensitive simulation was zero; as such, calculating fold change was not possible.

| **Node** | **Resistant**  **Score** | **Sensitive**  **Score** | **Fold Change (FC)** | **Log10 FC** | ***E_mod_*** | ***E_exp_*** | **ABS (*E_mod_-E_exp_*)** |
| --- | --- | --- | --- | --- | --- | --- | --- |
| **14-3-3** | 1111 | 1062 | 1.04613936 | 0.019589542 | 0 | 0 | 0 |
| **ABCA1** | 374 | 463 | 0.807775378 | -0.092709389 | 0 | 0 | 0 |
| **AFP** | 330 | 347 | 0.951008646 | -0.021815535 | 0 | 0 | 0 |
| **AP-1** | 20 | 529 | 0.037807183 | -1.422425676 | -1 | -1 | 0 |
| **ARHGAP35** | 496 | 557 | 0.89048474 | -0.050373519 | 0 | 0 | 0 |
| **BAG1** | 801 | 787 | 1.017789072 | 0.007657784 | 0 | 0 | 0 |
| **CD2** | 295 | 292 | 1.010273973 | 0.004439165 | 0 | 0 | 0 |
| **CD40LG** | 361 | 380 | 0.95 | -0.022276395 | 0 | 0 | 0 |
| **CREB1** | 983 | 1225 | 0.80244898 | -0.095582571 | 0 | 0 | 0 |
| **CREBBP/EP300** | 1355 | 1191 | 1.137699412 | 0.056027534 | 0 | 0 | 0 |
| **CRH** | 389 | 396 | 0.982323232 | -0.007745585 | 0 | 0 | 0 |
| **DAP3** | 908 | 858 | 1.058275058 | 0.024598561 | 0 | 0 | 0 |
| **DAXX** | 2138 | 1647 | 1.29811779 | 0.113314102 | 0 | 0 | 0 |
| **FSCN1** | 1154 | 887 | 1.301014656 | 0.114282189 | 0 | 1 | 1 |
| **GLUL** | 290 | 1119 | 0.259159964 | -0.586432089 | -1 | -1 | 0 |
| **GR** | 243 | 881 | 0.275822928 | -0.559369635 | -1 | -1 | 0 |
| **HDAC1** | 692 | 710 | 0.974647887 | -0.011152254 | 0 | 0 | 0 |
| **HDAC6** | 1117 | 1110 | 1.006306306 | 0.002730194 | 0 | 0 | 0 |
| **HSP90** | 3483 | 3173 | 1.097699338 | 0.040483402 | 0 | 0 | 0 |
| **IL10** | 618 | 1029 | 0.60058309 | -0.2214269 | 0 | -1 | 1 |
| **IL6** | 367 | 364 | 1.008241758 | 0.003564681 | 0 | 0 | 0 |
| **LIF** | 462 | 460 | 1.004347826 | 0.001884144 | 0 | 0 | 0 |
| **MED1** | 758 | 763 | 0.99344692 | -0.002855332 | 0 | 0 | 0 |
| **NCOA1** | 1179 | 1194 | 0.987437186 | -0.005490522 | 0 | -1 | 1 |
| **NCOA2** | 743 | 643 | 1.155520995 | 0.062777841 | 0 | 0 | 0 |
| **NCOA3** | 1180 | 1127 | 1.047027507 | 0.019958091 | 0 | 0 | 0 |
| **NCOA6** | 1486 | 1413 | 1.051663128 | 0.021876648 | 0 | 0 | 0 |
| **NCOR1** | 644 | 645 | 0.998449612 | -0.000673847 | 0 | 0 | 0 |
| **NCOR2** | 484 | 476 | 1.016806723 | 0.007238409 | 0 | 0 | 0 |
| **NFKB** | 338 | 442 | 0.764705882 | -0.116505569 | 0 | -1 | 1 |
| **NR1I3** | 290 | 299 | 0.969899666 | -0.01327319 | 0 | 0 | 0 |
| **NR2F2** | 243 | 261 | 0.931034483 | -0.031034234 | 0 | 0 | 0 |
| **NRIP1** | 838 | 847 | 0.989374262 | -0.004639392 | 0 | 0 | 0 |
| **PKA** | 1005 | 1138 | 0.883128295 | -0.0539762 | 0 | 0 | 0 |
| **POU2F1** | 510 | 528 | 0.965909091 | -0.015063746 | 0 | 0 | 0 |
| **PRKDC** | 2523 | 2316 | 1.089378238 | 0.037178695 | 0 | 0 | 0 |
| **PTGES3** | 2456 | 2280 | 1.077192982 | 0.032293515 | 0 | 0 | 0 |
| **SCAP** | 833 | 830 | 1.003614458 | 0.001566909 | 0 | 0 | 0 |
| **SGK1** | 986 | 1126 | 0.875666075 | -0.057661476 | 0 | -1 | 1 |
| **SMAD3** | 608 | 808 | 0.752475248 | -0.123507782 | 0 | -1 | 1 |
| **SMARCA4** | 891 | 818 | 1.089242054 | 0.0371244 | 0 | 1 | 1 |
| **STAT3** | 2712 | 2740 | 0.989781022 | -0.004460878 | 0 | 0 | 0 |
| **STAT5B** | 665 | 711 | 0.935302391 | -0.029047955 | 0 | 0 | 0 |
| **SUMO** | 1920 | 1728 | 1.111111111 | 0.045757491 | 0 | 0 | 0 |
| **TP53** | 105 | 0 | N/A | N/A | N/A | N/A | N/A |
| **TSC22D3** | 895 | 1112 | 0.804856115 | -0.094281752 | 0 | -1 | 1 |
| **UBC** | 1143 | 1191 | 0.959697733 | -0.017865531 | 0 | 0 | 0 |
|  | | | | | | | |
|  | | | **AVG** | -0.065376166 |  | **Correct** | 82.6% |
|  |  |  | **STDEV** | 0.242351648 |  | **Small Error** | 17.4% |
|  |  |  | **Upper** | 0.176975481 |  | **Large Error** | 0.0% |
|  |  |  | **Lower** | -0.307727814 |  |  |  |

**S13 Table:** Model validation by STSFA analysis (Comparison 2).

| **Node** | **Resistant**  **Score** | **Sensitive**  **Score** | **Fold Change (FC)** | **Log10 FC** | ***E_mod_*** | ***E_exp_*** | **ABS (*E_mod_-E_exp_*)** |
| --- | --- | --- | --- | --- | --- | --- | --- |
| **14-3-3** | 1096 | 1089 | 1.006427916 | 0.002782674 | 0 | 0 | 0 |
| **ABCA1** | 366 | 470 | 0.778723404 | -0.108616773 | 0 | -1 | 1 |
| **AFP** | 373 | 364 | 1.024725275 | 0.010607448 | 0 | 0 | 0 |
| **AP-1** | 3 | 330 | 0.009090909 | -2.041392685 | -1 | -1 | 0 |
| **ARHGAP35** | 533 | 535 | 0.996261682 | -0.001626573 | 0 | 0 | 0 |
| **BAG1** | 760 | 775 | 0.980645161 | -0.00848811 | 0 | 0 | 0 |
| **CD2** | 359 | 351 | 1.022792023 | 0.009787332 | 0 | 0 | 0 |
| **CD40LG** | 360 | 338 | 1.065088757 | 0.0273858 | 0 | 0 | 0 |
| **CREB1** | 971 | 959 | 1.012513034 | 0.005400623 | 0 | 1 | 1 |
| **CREBBP/EP300** | 1300 | 1072 | 1.212686567 | 0.083748567 | 0 | 0 | 0 |
| **CRH** | 433 | 425 | 1.018823529 | 0.008098966 | 0 | 0 | 0 |
| **DAP3** | 896 | 894 | 1.002237136 | 0.000970491 | 0 | 0 | 0 |
| **DAXX** | 2115 | 1791 | 1.180904523 | 0.072214786 | 0 | 0 | 0 |
| **FSCN1** | 1156 | 961 | 1.202913632 | 0.080234446 | 0 | 1 | 1 |
| **GLUL** | 316 | 1049 | 0.301239276 | -0.521088406 | -1 | -1 | 0 |
| **GR** | 65 | 506 | 0.128458498 | -0.89123716 | -1 | -1 | 0 |
| **HDAC1** | 694 | 728 | 0.953296703 | -0.020771909 | 0 | 0 | 0 |
| **HDAC6** | 1259 | 1132 | 1.112190813 | 0.046179303 | 0 | 0 | 0 |
| **HSP90** | 3479 | 3202 | 1.086508432 | 0.036033101 | 0 | 0 | 0 |
| **IL10** | 655 | 779 | 0.840821566 | -0.075296158 | 0 | -1 | 1 |
| **IL6** | 374 | 335 | 1.11641791 | 0.047826795 | 0 | 0 | 0 |
| **LIF** | 488 | 439 | 1.111617312 | 0.045955302 | 0 | 0 | 0 |
| **MED1** | 754 | 747 | 1.009370817 | 0.004050744 | 0 | 0 | 0 |
| **NCOA1** | 1094 | 1058 | 1.034026465 | 0.014531654 | 0 | 0 | 0 |
| **NCOA2** | 652 | 591 | 1.10321489 | 0.042660115 | 0 | 0 | 0 |
| **NCOA3** | 1101 | 934 | 1.178800857 | 0.071440443 | 0 | 1 | 1 |
| **NCOA6** | 1488 | 1393 | 1.068198134 | 0.028651815 | 0 | 0 | 0 |
| **NCOR1** | 638 | 674 | 0.946587537 | -0.023839218 | 0 | 0 | 0 |
| **NCOR2** | 496 | 490 | 1.012244898 | 0.005285596 | 0 | 0 | 0 |
| **NFKB** | 306 | 344 | 0.889534884 | -0.050837016 | 0 | 0 | 0 |
| **NR1I3** | 267 | 303 | 0.881188119 | -0.054931367 | 0 | 0 | 0 |
| **NR2F2** | 250 | 250 | 1 | 0 | 0 | 0 | 0 |
| **NRIP1** | 832 | 751 | 1.107856192 | 0.044483389 | 0 | 1 | 1 |
| **PKA** | 1036 | 1110 | 0.933333333 | -0.029963223 | 0 | 0 | 0 |
| **POU2F1** | 491 | 533 | 0.92120075 | -0.035645717 | 0 | 0 | 0 |
| **PRKDC** | 2521 | 2362 | 1.067315834 | 0.028292952 | 0 | 0 | 0 |
| **PTGES3** | 2425 | 2359 | 1.027977957 | 0.011983802 | 0 | 0 | 0 |
| **SCAP** | 806 | 831 | 0.969915764 | -0.013265982 | 0 | 0 | 0 |
| **SGK1** | 980 | 894 | 1.096196868 | 0.039888557 | 0 | 0 | 0 |
| **SMAD3** | 608 | 685 | 0.887591241 | -0.051786992 | 0 | -1 | 1 |
| **SMARCA4** | 867 | 846 | 1.024822695 | 0.010648734 | 0 | 0 | 0 |
| **STAT3** | 2564 | 2386 | 1.074601844 | 0.031247582 | 0 | 0 | 0 |
| **STAT5B** | 662 | 690 | 0.95942029 | -0.017991101 | 0 | 0 | 0 |
| **SUMO** | 1915 | 1877 | 1.020245072 | 0.008704506 | 0 | -1 | 1 |
| **TP53** | 141 | 138 | 1.02173913 | 0.009340026 | 0 | 0 | 0 |
| **TSC22D3** | 886 | 959 | 0.923879041 | -0.034384885 | 0 | 0 | 0 |
| **UBC** | 1146 | 1131 | 1.013262599 | 0.005722013 | 0 | 0 | 0 |
|  | | | | | | | |
|  | | | **AVG** | -0.066957568 |  | **Correct** | 83.0% |
|  |  |  | **STDEV** | 0.332789223 |  | **Small Error** | 17.0% |
|  |  |  | **Upper** | 0.265831655 |  | **Large Error** | 0.0% |
|  |  |  | **Lower** | -0.399746791 |  |  |  |

**S14 Table:** Model validation by STSFA analysis (Comparison 3).

| **Node** | **Resistant**  **Score** | **Sensitive**  **Score** | **Fold Change (FC)** | **Log10 FC** | ***E_mod_*** | ***E_exp_*** | **ABS (*E_mod_-E_exp_*)** |
| --- | --- | --- | --- | --- | --- | --- | --- |
| **14-3-3** | 1091 | 1104 | 0.988224638 | -0.005144323 | 0 | 0 | 0 |
| **ABCA1** | 382 | 379 | 1.007915567 | 0.003424153 | 0 | 0 | 0 |
| **AFP** | 358 | 349 | 1.025787966 | 0.0110576 | 0 | 0 | 0 |
| **AP-1** | 181 | 368 | 0.491847826 | -0.308169244 | -1 | -1 | 0 |
| **ARHGAP35** | 546 | 553 | 0.987341772 | -0.005532489 | 0 | 0 | 0 |
| **BAG1** | 806 | 776 | 1.038659794 | 0.016473321 | 0 | 0 | 0 |
| **CD2** | 318 | 355 | 0.895774648 | -0.047801233 | 0 | 0 | 0 |
| **CD40LG** | 372 | 401 | 0.927680798 | -0.032601433 | 0 | 0 | 0 |
| **CREB1** | 899 | 868 | 1.035714286 | 0.015239967 | 0 | 0 | 0 |
| **CREBBP/EP300** | 1352 | 1169 | 1.156544055 | 0.06316218 | 0 | 0 | 0 |
| **CRH** | 437 | 417 | 1.047961631 | 0.020345382 | 0 | 0 | 0 |
| **DAP3** | 894 | 905 | 0.987845304 | -0.00531106 | 0 | 0 | 0 |
| **DAXX** | 2169 | 2052 | 1.057017544 | 0.024082196 | 0 | 0 | 0 |
| **FSCN1** | 1250 | 1062 | 1.177024482 | 0.070785496 | 0 | 1 | 1 |
| **GLUL** | 302 | 942 | 0.32059448 | -0.49404396 | -1 | -1 | 0 |
| **GR** | 65 | 171 | 0.380116959 | -0.420082754 | -1 | -1 | 0 |
| **HDAC1** | 684 | 739 | 0.925575101 | -0.033588337 | 0 | 0 | 0 |
| **HDAC6** | 1215 | 1249 | 0.972778223 | -0.01198616 | 0 | 0 | 0 |
| **HSP90** | 3614 | 3520 | 1.026704545 | 0.011445485 | 0 | 0 | 0 |
| **IL10** | 750 | 706 | 1.062322946 | 0.026256562 | 0 | 0 | 0 |
| **IL6** | 412 | 429 | 0.96037296 | -0.017560076 | 0 | 0 | 0 |
| **LIF** | 481 | 483 | 0.995859213 | -0.001802054 | 0 | 0 | 0 |
| **MED1** | 749 | 726 | 1.031680441 | 0.013545197 | 0 | 0 | 0 |
| **NCOA1** | 1204 | 1115 | 1.079820628 | 0.03335162 | 0 | 0 | 0 |
| **NCOA2** | 657 | 595 | 1.104201681 | 0.043048404 | 0 | 0 | 0 |
| **NCOA3** | 1129 | 991 | 1.13925328 | 0.056620287 | 0 | 1 | 1 |
| **NCOA6** | 1534 | 1433 | 1.070481507 | 0.029579169 | 0 | 0 | 0 |
| **NCOR1** | 663 | 660 | 1.004545455 | 0.001969593 | 0 | 0 | 0 |
| **NCOR2** | 513 | 486 | 1.055555556 | 0.023481096 | 0 | 0 | 0 |
| **NFKB** | 321 | 343 | 0.935860058 | -0.028789088 | 0 | 0 | 0 |
| **NR1I3** | 272 | 289 | 0.941176471 | -0.026328939 | 0 | 0 | 0 |
| **NR2F2** | 226 | 256 | 0.8828125 | -0.054131526 | 0 | 0 | 0 |
| **NRIP1** | 862 | 765 | 1.126797386 | 0.051845831 | 0 | 1 | 1 |
| **PKA** | 1083 | 1091 | 0.992667278 | -0.003196294 | 0 | 0 | 0 |
| **POU2F1** | 502 | 504 | 0.996031746 | -0.001726819 | 0 | 0 | 0 |
| **PRKDC** | 2654 | 2504 | 1.059904153 | 0.025266594 | 0 | 0 | 0 |
| **PTGES3** | 2498 | 2474 | 1.009700889 | 0.004192739 | 0 | 0 | 0 |
| **SCAP** | 842 | 825 | 1.020606061 | 0.008858143 | 0 | 0 | 0 |
| **SGK1** | 766 | 700 | 1.094285714 | 0.03913073 | 0 | 0 | 0 |
| **SMAD3** | 639 | 694 | 0.92074928 | -0.035858612 | 0 | -1 | 1 |
| **SMARCA4** | 938 | 855 | 1.097076023 | 0.040236724 | 0 | 1 | 1 |
| **STAT3** | 2762 | 2617 | 1.055406955 | 0.023419952 | 0 | 0 | 0 |
| **STAT5B** | 644 | 679 | 0.948453608 | -0.022983907 | 0 | 0 | 0 |
| **SUMO** | 1949 | 1996 | 0.976452906 | -0.010348698 | 0 | -1 | 1 |
| **TP53** | 130 | 143 | 0.909090909 | -0.041392685 | 0 | 0 | 0 |
| **TSC22D3** | 506 | 535 | 0.945794393 | -0.024203265 | 0 | 0 | 0 |
| **UBC** | 1139 | 1125 | 1.012444444 | 0.005371202 | 0 | 0 | 0 |
|  | | | | | | | |
|  | | | **AVG** | -0.020646667 |  | **Correct** | 87.2% |
|  |  |  | **STDEV** | 0.107902042 |  | **Small Error** | 12.8% |
|  |  |  | **Upper** | 0.087255375 |  | **Large Error** | 0.0% |
|  |  |  | **Lower** | -0.128548708 |  |  |  |

**S15 Table:** Model validation by STSFA analysis (Comparison 4).

| **Node** | **Resistant**  **Score** | **Sensitive**  **Score** | **Fold Change (FC)** | **Log10 FC** | ***E_mod_*** | ***E_exp_*** | **ABS (*E_mod_-E_exp_*)** |
| --- | --- | --- | --- | --- | --- | --- | --- |
| **14-3-3** | 1054 | 1099 | 0.959053685 | -0.018157082 | 0 | 0 | 0 |
| **ABCA1** | 692 | 472 | 1.466101695 | 0.166164096 | 1 | 1 | 0 |
| **AFP** | 346 | 395 | 0.875949367 | -0.057520997 | 0 | -1 | 1 |
| **AP-1** | 151 | 111 | 1.36036036 | 0.133653969 | 1 | 0 | 1 |
| **ARHGAP35** | 520 | 528 | 0.984848485 | -0.006630579 | 0 | 0 | 0 |
| **BAG1** | 828 | 805 | 1.028571429 | 0.012234456 | 0 | 0 | 0 |
| **CD2** | 343 | 304 | 1.128289474 | 0.052420536 | 0 | 0 | 0 |
| **CD40LG** | 375 | 401 | 0.935162095 | -0.029113105 | 0 | 0 | 0 |
| **CREB1** | 1330 | 1215 | 1.094650206 | 0.039275363 | 0 | 0 | 0 |
| **CREBBP/EP300** | 1456 | 1386 | 1.050505051 | 0.021398145 | 0 | 0 | 0 |
| **CRH** | 395 | 422 | 0.936018957 | -0.028715355 | 0 | 0 | 0 |
| **DAP3** | 914 | 853 | 1.071512309 | 0.029997165 | 0 | 1 | 1 |
| **DAXX** | 1898 | 1920 | 0.988541667 | -0.005005021 | 0 | 0 | 0 |
| **FSCN1** | 664 | 1017 | 0.652900688 | -0.185152874 | -1 | -1 | 0 |
| **GLUL** | 952 | 1014 | 0.938856016 | -0.027401007 | 0 | -1 | 1 |
| **GR** | 379 | 153 | 2.477124183 | 0.393947779 | 1 | -1 | 2 |
| **HDAC1** | 643 | 695 | 0.925179856 | -0.033773832 | 0 | 0 | 0 |
| **HDAC6** | 1178 | 1167 | 1.009425878 | 0.004074434 | 0 | 0 | 0 |
| **HSP90** | 3462 | 3258 | 1.062615101 | 0.026375984 | 0 | 0 | 0 |
| **IL10** | 805 | 724 | 1.111878453 | 0.046057314 | 0 | 0 | 0 |
| **IL6** | 273 | 340 | 0.802941176 | -0.09531627 | -1 | 0 | 1 |
| **LIF** | 429 | 516 | 0.831395349 | -0.080192409 | 0 | -1 | 1 |
| **MED1** | 815 | 715 | 1.13986014 | 0.056851567 | 0 | 1 | 1 |
| **NCOA1** | 1225 | 1307 | 0.937260903 | -0.028139499 | 0 | -1 | 1 |
| **NCOA2** | 860 | 810 | 1.061728395 | 0.026013432 | 0 | 0 | 0 |
| **NCOA3** | 1185 | 1168 | 1.014554795 | 0.006275508 | 0 | 0 | 0 |
| **NCOA6** | 1308 | 1268 | 1.031545741 | 0.01348849 | 0 | 0 | 0 |
| **NCOR1** | 613 | 643 | 0.953343701 | -0.020750498 | 0 | 0 | 0 |
| **NCOR2** | 431 | 479 | 0.899791232 | -0.045858243 | 0 | 0 | 0 |
| **NFKB** | 336 | 408 | 0.823529412 | -0.084320886 | -1 | -1 | 0 |
| **NR1I3** | 269 | 294 | 0.914965986 | -0.03859505 | 0 | 0 | 0 |
| **NR2F2** | 278 | 250 | 1.112 | 0.046104787 | 0 | 0 | 0 |
| **NRIP1** | 1097 | 1121 | 0.978590544 | -0.009398985 | 0 | 0 | 0 |
| **PKA** | 1201 | 1158 | 1.037132988 | 0.015834448 | 0 | 0 | 0 |
| **POU2F1** | 502 | 523 | 0.959847036 | -0.017797972 | 0 | 0 | 0 |
| **PRKDC** | 2473 | 2356 | 1.049660441 | 0.02104883 | 0 | 0 | 0 |
| **PTGES3** | 2436 | 2304 | 1.057291667 | 0.024194809 | 0 | 0 | 0 |
| **SCAP** | 719 | 775 | 0.927741935 | -0.032572812 | 0 | -1 | 1 |
| **SGK1** | 1110 | 1097 | 1.011850501 | 0.005116351 | 0 | 1 | 1 |
| **SMAD3** | 1040 | 969 | 1.073271414 | 0.030709562 | 0 | 0 | 0 |
| **SMARCA4** | 878 | 929 | 0.94510226 | -0.024521198 | 0 | 0 | 0 |
| **STAT3** | 2437 | 2626 | 0.928027418 | -0.032439193 | 0 | -1 | 1 |
| **STAT5B** | 649 | 647 | 1.00309119 | 0.001340416 | 0 | 0 | 0 |
| **SUMO** | 1760 | 1705 | 1.032258065 | 0.013788284 | 0 | 0 | 0 |
| **TP53** | 74 | 100 | 0.74 | -0.13076828 | -1 | -1 | 0 |
| **TSC22D3** | 718 | 839 | 0.855780691 | -0.067637517 | 0 | -1 | 1 |
| **UBC** | 1110 | 1097 | 1.011850501 | 0.005116351 | 0 | 0 | 0 |
|  | | | | | | | |
|  | | | **AVG** | 0.001951137 |  | **Correct** | 72.3% |
|  |  |  | **STDEV** | 0.082308007 |  | **Small Error** | 25.5% |
|  |  |  | **Upper** | 0.084259144 |  | **Large Error** | 2.1% |
|  |  |  | **Lower** | -0.080356871 |  |  |  |

**S16 Table:** Model validation by STSFA analysis (Comparison 5).

| **Node** | **Resistant**  **Score** | **Sensitive**  **Score** | **Fold Change (FC)** | **Log10 FC** | ***E_mod_*** | ***E_exp_*** | **ABS (*E_mod_-E_exp_*)** |
| --- | --- | --- | --- | --- | --- | --- | --- |
| **14-3-3** | 1055 | 1102 | 0.957350272 | -0.018929135 | 0 | 0 | 0 |
| **ABCA1** | 649 | 437 | 1.485125858 | 0.17176326 | 1 | 1 | 0 |
| **AFP** | 410 | 325 | 1.261538462 | 0.100900496 | 1 | 1 | 0 |
| **AP-1** | 123 | 110 | 1.118181818 | 0.048512426 | 0 | 0 | 0 |
| **ARHGAP35** | 500 | 504 | 0.992063492 | -0.003460532 | 0 | 0 | 0 |
| **BAG1** | 804 | 801 | 1.003745318 | 0.001623533 | 0 | 0 | 0 |
| **CD2** | 294 | 280 | 1.05 | 0.021189299 | 0 | 0 | 0 |
| **CD40LG** | 371 | 358 | 1.036312849 | 0.015490883 | 0 | 0 | 0 |
| **CREB1** | 1353 | 1203 | 1.124688279 | 0.051032169 | 0 | 0 | 0 |
| **CREBBP/EP300** | 1465 | 1429 | 1.025192442 | 0.010805396 | 0 | 0 | 0 |
| **CRH** | 397 | 395 | 1.005063291 | 0.002193411 | 0 | 0 | 0 |
| **DAP3** | 924 | 887 | 1.041713641 | 0.017748351 | 0 | 1 | 1 |
| **DAXX** | 1917 | 1990 | 0.963316583 | -0.016230964 | 0 | -1 | 1 |
| **FSCN1** | 701 | 938 | 0.747334755 | -0.12648482 | -1 | -1 | 0 |
| **GLUL** | 972 | 1016 | 0.956692913 | -0.019227443 | 0 | -1 | 1 |
| **GR** | 429 | 171 | 2.50877193 | 0.399461182 | 1 | -1 | 2 |
| **HDAC1** | 646 | 680 | 0.95 | -0.022276395 | 0 | 0 | 0 |
| **HDAC6** | 1102 | 1142 | 0.96497373 | -0.015484509 | 0 | 0 | 0 |
| **HSP90** | 3472 | 3323 | 1.044839001 | 0.019049375 | 0 | 0 | 0 |
| **IL10** | 734 | 670 | 1.095522388 | 0.039621257 | 0 | 0 | 0 |
| **IL6** | 266 | 342 | 0.777777778 | -0.109144469 | -1 | 0 | 1 |
| **LIF** | 416 | 471 | 0.883227176 | -0.053927577 | 0 | 0 | 0 |
| **MED1** | 815 | 769 | 1.059817945 | 0.025231269 | 0 | 0 | 0 |
| **NCOA1** | 1217 | 1191 | 1.021830395 | 0.009378817 | 0 | 0 | 0 |
| **NCOA2** | 863 | 854 | 1.010538642 | 0.004552925 | 0 | 0 | 0 |
| **NCOA3** | 1182 | 1161 | 1.018087855 | 0.007785257 | 0 | 0 | 0 |
| **NCOA6** | 1277 | 1287 | 0.992229992 | -0.00338765 | 0 | 0 | 0 |
| **NCOR1** | 578 | 641 | 0.901716069 | -0.044930191 | 0 | -1 | 1 |
| **NCOR2** | 437 | 490 | 0.891836735 | -0.049714643 | 0 | -1 | 1 |
| **NFKB** | 313 | 377 | 0.830238727 | -0.080797013 | 0 | -1 | 1 |
| **NR1I3** | 293 | 282 | 1.039007092 | 0.016618512 | 0 | 0 | 0 |
| **NR2F2** | 231 | 244 | 0.946721311 | -0.023777846 | 0 | 0 | 0 |
| **NRIP1** | 1062 | 1119 | 0.949061662 | -0.02270557 | 0 | -1 | 1 |
| **PKA** | 1216 | 1076 | 1.130111524 | 0.053121304 | 0 | 0 | 0 |
| **POU2F1** | 499 | 492 | 1.014227642 | 0.006135443 | 0 | 0 | 0 |
| **PRKDC** | 2464 | 2495 | 0.98757515 | -0.005429846 | 0 | -1 | 1 |
| **PTGES3** | 2465 | 2372 | 1.03920742 | 0.016702239 | 0 | 0 | 0 |
| **SCAP** | 724 | 757 | 0.956406869 | -0.019357313 | 0 | -1 | 1 |
| **SGK1** | 1194 | 1176 | 1.015306122 | 0.006597005 | 0 | 0 | 0 |
| **SMAD3** | 941 | 972 | 0.968106996 | -0.014076641 | 0 | 0 | 0 |
| **SMARCA4** | 854 | 916 | 0.93231441 | -0.030437603 | 0 | -1 | 1 |
| **STAT3** | 2439 | 2397 | 1.017521902 | 0.007543766 | 0 | 0 | 0 |
| **STAT5B** | 630 | 650 | 0.969230769 | -0.013572807 | 0 | 0 | 0 |
| **SUMO** | 1790 | 1792 | 0.998883929 | -0.000484974 | 0 | 0 | 0 |
| **TP53** | 41 | 117 | 0.35042735 | -0.455402005 | -1 | -1 | 0 |
| **TSC22D3** | 779 | 784 | 0.993622449 | -0.002778605 | 0 | 0 | 0 |
| **UBC** | 1108 | 1101 | 1.006357856 | 0.002752441 | 0 | 0 | 0 |
|  | | | | | | | |
|  | | | **AVG** | -0.00204699 |  | **Correct** | 74.5% |
|  |  |  | **STDEV** | 0.100561938 |  | **Small Error** | 23.4% |
|  |  |  | **Upper** | 0.098514947 |  | **Large Error** | 2.1% |
|  |  |  | **Lower** | -0.102608928 |  |  |  |

**S17 Table:** Model validation by STSFA analysis (Comparison 6).

| **Node** | **Resistant**  **Score** | **Sensitive**  **Score** | **Fold Change (FC)** | **Log10 FC** | ***E_mod_*** | ***E_exp_*** | **ABS (*E_mod_-E_exp_*)** |
| --- | --- | --- | --- | --- | --- | --- | --- |
| **14-3-3** | 1063 | 1082 | 0.982439926 | -0.007693996 | 0 | 0 | 0 |
| **ABCA1** | 722 | 457 | 1.579868709 | 0.198620997 | 1 | 1 | 0 |
| **AFP** | 337 | 344 | 0.979651163 | -0.008928542 | 0 | 0 | 0 |
| **AP-1** | 199 | 218 | 0.912844037 | -0.039603417 | 0 | 0 | 0 |
| **ARHGAP35** | 526 | 515 | 1.021359223 | 0.009178515 | 0 | 0 | 0 |
| **BAG1** | 801 | 812 | 0.986453202 | -0.005923513 | 0 | 0 | 0 |
| **CD2** | 312 | 311 | 1.003215434 | 0.001394205 | 0 | 0 | 0 |
| **CD40LG** | 389 | 370 | 1.051351351 | 0.021747877 | 0 | 0 | 0 |
| **CREB1** | 1298 | 1234 | 1.051863857 | 0.021959533 | 0 | 0 | 0 |
| **CREBBP/EP300** | 1509 | 1481 | 1.018906144 | 0.008134181 | 0 | 0 | 0 |
| **CRH** | 389 | 409 | 0.951100244 | -0.021773707 | 0 | 0 | 0 |
| **DAP3** | 926 | 887 | 1.043968433 | 0.018687367 | 0 | 1 | 1 |
| **DAXX** | 1956 | 2009 | 0.973618716 | -0.011611086 | 0 | 0 | 0 |
| **FSCN1** | 672 | 1010 | 0.665346535 | -0.176952101 | -1 | -1 | 0 |
| **GLUL** | 956 | 951 | 1.005257624 | 0.002277375 | 0 | 0 | 0 |
| **GR** | 560 | 196 | 2.857142857 | 0.455931956 | 1 | -1 | 2 |
| **HDAC1** | 629 | 668 | 0.941616766 | -0.026125817 | 0 | 0 | 0 |
| **HDAC6** | 1138 | 1121 | 1.015165031 | 0.006536649 | 0 | 0 | 0 |
| **HSP90** | 3570 | 3442 | 1.037187682 | 0.01585735 | 0 | 0 | 0 |
| **IL10** | 976 | 752 | 1.29787234 | 0.113231977 | 1 | 1 | 0 |
| **IL6** | 234 | 351 | 0.666666667 | -0.176091259 | -1 | 0 | 1 |
| **LIF** | 401 | 453 | 0.885209713 | -0.052953829 | 0 | 0 | 0 |
| **MED1** | 848 | 817 | 1.037943696 | 0.016173796 | 0 | 0 | 0 |
| **NCOA1** | 1242 | 1213 | 1.023907667 | 0.010260795 | 0 | 0 | 0 |
| **NCOA2** | 876 | 896 | 0.977678571 | -0.009803903 | 0 | 0 | 0 |
| **NCOA3** | 1223 | 1207 | 1.013256007 | 0.005719187 | 0 | 0 | 0 |
| **NCOA6** | 1360 | 1361 | 0.999265246 | -0.000319217 | 0 | 0 | 0 |
| **NCOR1** | 590 | 652 | 0.904907975 | -0.043395584 | 0 | -1 | 1 |
| **NCOR2** | 467 | 454 | 1.028634361 | 0.012261028 | 0 | 0 | 0 |
| **NFKB** | 329 | 384 | 0.856770833 | -0.067135326 | 0 | 0 | 0 |
| **NR1I3** | 266 | 283 | 0.939929329 | -0.026904799 | 0 | 0 | 0 |
| **NR2F2** | 245 | 235 | 1.042553191 | 0.018098222 | 0 | 0 | 0 |
| **NRIP1** | 1087 | 1142 | 0.951838879 | -0.02143656 | 0 | -1 | 1 |
| **PKA** | 1256 | 1112 | 1.129496403 | 0.052884852 | 0 | 0 | 0 |
| **POU2F1** | 512 | 511 | 1.001956947 | 0.000849061 | 0 | 0 | 0 |
| **PRKDC** | 2559 | 2591 | 0.987649556 | -0.005397127 | 0 | -1 | 1 |
| **PTGES3** | 2512 | 2446 | 1.026982829 | 0.011563182 | 0 | 0 | 0 |
| **SCAP** | 735 | 786 | 0.935114504 | -0.029135207 | 0 | -1 | 1 |
| **SGK1** | 1061 | 1094 | 0.969835466 | -0.013301938 | 0 | 0 | 0 |
| **SMAD3** | 960 | 934 | 1.027837259 | 0.011924357 | 0 | 0 | 0 |
| **SMARCA4** | 865 | 928 | 0.932112069 | -0.030531869 | 0 | -1 | 1 |
| **STAT3** | 2517 | 2492 | 1.010032103 | 0.004335178 | 0 | 0 | 0 |
| **STAT5B** | 644 | 678 | 0.949852507 | -0.022343827 | 0 | -1 | 1 |
| **SUMO** | 1819 | 1817 | 1.001100715 | 0.000477772 | 0 | 0 | 0 |
| **TP53** | 61 | 91 | 0.67032967 | -0.173711557 | -1 | -1 | 0 |
| **TSC22D3** | 489 | 511 | 0.956947162 | -0.019112041 | 0 | 0 | 0 |
| **UBC** | 1126 | 1107 | 1.017163505 | 0.00739077 | 0 | 0 | 0 |
|  | | | | | | | |
|  | | | **AVG** | 0.000751276 |  | **Correct** | 80.9% |
|  |  |  | **STDEV** | 0.090259932 |  | **Small Error** | 17.0% |
|  |  |  | **Upper** | 0.091011208 |  | **Large Error** | 2.1% |
|  |  |  | **Lower** | -0.089508657 |  |  |  |

**S18 Table:** Systematically incorrect nodes for the comparisons shown in Table 8. Among the most commonly incorrect nodes were SUMO, DAXX, and MED1. SUMOylation is worth studying in leukaemia as it is understudied whilst DAXX is linked to SUMO which may explain its incorrect prediction. MED1 is a general transcription factor for RNA polymerase II, which again may explain its incorrect prediction as such a general transcription factor is unlikely to be well simulated by a small model.

| **Node** | **Comparison** | | | | | | **Total** | **Incorrect?** | | |
| --- | --- | --- | --- | --- | --- | --- | --- | --- | --- | --- |
|  | **1** | **2** | **3** | **4** | **5** | **6** |  |  |  |  |
| **14-3-3.** | 0 | 0 | 0 | 0 | 0 | 0 | 0 | NO | | |
| **ABCA1** | 0 | 1 | 0 | 1 | 1 | 1 | 4 | YES | | |
| **AFP** | 1 | 1 | 1 | 0 | 2 | 1 | 6 | YES | | |
| **AP-1** | 1 | 1 | 1 | 0 | 0 | 0 | 3 | NO | | |
| **ARHGAP35** | 1 | 1 | 1 | 1 | 1 | 1 | 6 | YES | | |
| **BAG1** | 0 | 0 | 0 | 0 | 0 | 0 | 0 | NO | | |
| **CD2** | 0 | 0 | 0 | 0 | 0 | 0 | 0 | NO | | |
| **CD40LG** | 1 | 1 | 1 | 1 | 1 | 1 | 6 | YES | | |
| **CREB1** | 0 | 1 | 0 | 0 | 0 | 0 | 1 | NO | | |
| **CREBBP/EP300** | 0 | 0 | 0 | 0 | 0 | 0 | 0 | NO | | |
| **CRH** | 0 | 0 | 0 | 0 | 0 | 0 | 0 | NO | | |
| **DAP3** | 0 | 0 | 0 | 1 | 1 | 1 | 3 | NO | | |
| **DAXX** | 1 | 1 | 1 | 1 | 2 | 1 | 7 | YES | | |
| **FSCN1** | 1 | 1 | 1 | 1 | 1 | 1 | 6 | YES | | |
| **GLUL** | 0 | 0 | 0 | 0 | 0 | 1 | 1 | NO | | |
| **HDAC1** | 1 | 1 | 1 | 1 | 1 | 1 | 6 | YES | | |
| **HDAC6** | 0 | 0 | 0 | 0 | 0 | 0 | 0 | NO | | |
| **HSP90** | 0 | 0 | 0 | 0 | 0 | 0 | 0 | NO | | |
| **IL10** | 1 | 1 | 0 | 0 | 0 | 1 | 3 | NO | | |
| **IL6** | 0 | 0 | 0 | 0 | 0 | 0 | 0 | NO | | |
| **LIF** | 0 | 0 | 0 | 1 | 0 | 0 | 1 | NO | | |
| **MED1** | 1 | 1 | 1 | 2 | 1 | 1 | 7 | YES | | |
| **NCOA1** | 1 | 0 | 0 | 1 | 0 | 0 | 2 | NO | | |
| **NCOA2** | 0 | 0 | 0 | 0 | 0 | 0 | 0 | NO | | |
| **NCOA3** | 0 | 1 | 1 | 0 | 0 | 0 | 2 | NO | | |
| **NCOA6** | 0 | 0 | 0 | 0 | 0 | 0 | 0 | NO | | |
| **NCOR1** | 0 | 0 | 0 | 0 | 1 | 1 | 2 | NO | | |
| **NCOR2** | 0 | 0 | 0 | 0 | 1 | 0 | 1 | NO | | |
| **NFKB** | 1 | 0 | 0 | 1 | 1 | 0 | 3 | NO | | |
| **NR1I3** | 1 | 1 | 1 | 1 | 1 | 1 | 6 | YES | | |
| **NR2F2** | 1 | 1 | 1 | 1 | 1 | 1 | 6 | YES | | |
| **NRIP1** | 0 | 1 | 1 | 0 | 1 | 1 | 4 | YES | | |
| **PKA** | 0 | 0 | 0 | 0 | 0 | 0 | 0 | NO | | |
| **POU2F1** | 0 | 0 | 0 | 0 | 0 | 0 | 0 | NO | | |
| **POU2F2** | 0 | 1 | 0 | 1 | 0 | 0 | 2 | NO | | |
| **PRKDC** | 0 | 0 | 0 | 0 | 1 | 1 | 2 | NO | | |
| **PTGES3** | 0 | 0 | 0 | 0 | 0 | 0 | 0 | NO | | |
| **SCAP** | 1 | 1 | 1 | 0 | 0 | 0 | 3 | NO | | |
| **SGK1** | 1 | 0 | 0 | 1 | 0 | 0 | 2 | NO | | |
| **SMAD3** | 1 | 1 | 1 | 0 | 0 | 0 | 3 | NO | | |
| **SMARCA4** | 1 | 0 | 1 | 0 | 1 | 1 | 4 | YES | | |
| **STAT3** | 0 | 0 | 0 | 1 | 0 | 0 | 1 | NO | | |
| **STAT5B** | 1 | 1 | 1 | 1 | 1 | 0 | 5 | YES | | |
| **SUMO** | 1 | 2 | 2 | 1 | 1 | 1 | 8 | YES | | |
| **TP53** | 0 | 0 | 0 | 1 | 1 | 1 | 3 | NO | | |
| **TSC22D3** | 0 | 1 | 1 | 0 | 1 | 1 | 4 | YES | | |
| **TSG101** | 0 | 0 | 0 | 0 | 0 | 1 | 1 | NO | | |
| **UBC** | 1 | 1 | 1 | 1 | 1 | 1 | 6 | YES | | |
|  | | | | | | | | | | |
|  | | | | | | **Correct** | | | 32 | 66.67 |
|  |  |  |  |  |  | **Incorrect** | | | 16 | 33.33 |
